# Germicidal UV Light and Incidence of Acute Respiratory Infection in Long-Term Care for Older Adults: A Randomized Clinical Trial

**DOI:** 10.1001/jamainternmed.2025.3388

**Published:** 2025-07-28

**Authors:** Andrew P. Shoubridge, Amanda Brass, Maria Crotty, Lidia Morawska, Scott C. Bell, Erin Flynn, Caroline Miller, Yiming Wang, Carol A. Holden, Megan Corlis, Nicolas Larby, Paul Worley, Levi Elms, Sarah K. Manning, Ming Qiao, Maria C. Inacio, Steve L. Wesselingh, Lito E. Papanicolas, Richard J. Woodman, Steven L. Taylor, Geraint B. Rogers

**Affiliations:** 1 South Australian Health and Medical Research Institute, Adelaide, South Australia, Australia; 2 College of Medicine and Public Health, Flinders University, Adelaide, South Australia, Australia; 3 Southern Adelaide Local Health Network, SA Health, Adelaide, South Australia, Australia; 4 International Laboratory for Air Quality and Health, Queensland University of Technology, Brisbane, Queensland, Australia; 5 The Prince Charles Hospital, Brisbane, Queensland, Australia; 6 Child Health Research Centre, Faculty of Medicine, University of Queensland, Brisbane, Queensland, Australia; 7 SA Pathology, SA Health, Adelaide, South Australia, Australia; 8 School of Public Health, University of Adelaide, Adelaide, South Australia, Australia; 9 Australian Nursing and Midwifery Federation, South Australia, Australia; 10 Aged Care Property Services Management, South Australia, Australia; 11 Riverland Mallee Coorong Local Health Network, Murray Bridge, South Australia, Australia; 12 Registry of Senior Australians Research Centre, South Australian Health and Medical Research Institute, Adelaide, South Australia, Australia; 13 Registry of Senior Australians Research Centre, Caring Futures Institute, College of Nursing and Health Services, Flinders University, Bedford Park, South Australia, Australia

## Abstract

**Importance:**

Infectious outbreaks of respiratory viruses within long-term care facilities (LTCFs) for older adults are associated with high rates of hospitalization and death. Despite evidence that airborne transmission contributes substantially to the spread of respiratory viruses within residential care for older adults, this mode of transmission has been largely unaddressed by existing infection control practices.

**Objective:**

To determine whether germicidal UV (GUV) appliances reduce acute respiratory infection (ARI) incidence in LTCFs.

**Design, Setting, and Participants:**

This multicenter, 2-arm, double-crossover, cluster randomized clinical trial assessed the effectiveness of GUV appliances in common spaces on the incidence of ARIs in 4 LTCFs in metropolitan and regional South Australia. LTCFs were divided into 2 equally sized zones (mean [SD] size, 44 [9] beds per zone). Within each LTCF, zones were randomized to active GUV appliances (intervention) or inactive (control) for 6 weeks, which was followed by a 2-week washout, crossover, and a further 2-week washout. Seven consecutive cycles were performed during the 110-week study period from August 31, 2021, to November 13, 2023. Data were analyzed from January 18, 2024, to December 4, 2024.

**Intervention:**

Continuous GUV appliance activity within common (non–resident room) areas for 6 weeks.

**Main Outcome and Measures:**

The primary outcome was the incidence rate of ARIs (per zone per cycle). A secondary analysis of long-term trends was performed based on infections per week.

**Results:**

Eight assessed zones across 4 LTCFs represented a total of 211 952 bed-days. Of 596 ARIs recorded across all zones, 475 (79.7%) occurred during intervention or control periods. The incidence rate in the control arm was 4.17 infections per zone per cycle (95% CI, 2.43-5.91), compared with 3.81 infections per zone per cycle (95% CI, 2.21-5.41) in the intervention arm (incidence rate ratio, 0.91; 95% CI, 0.77-1.09; *P* = .33). A posteriori secondary analysis with time-series autoregressive modeling showed that the control group recorded 2.61 ARIs per week (95% CI, 2.51-2.70) compared with 2.29 ARIs per week (95% CI, 2.06-2.51) in the intervention group (mean difference, 0.32; 95% CI, 0.10-0.54; *P* = .004).

**Conclusions and Relevance:**

This randomized clinical trial found that GUV light appliances in common areas of LTCFs did not reduce the incidence rate of ARIs per zone per cycle but did modestly reduce the total numbers of ARIs by the study conclusion. GUV appliances might be considered to support existing infection prevention and control practices in these settings.

**Trial Registration:**

Australian and New Zealand Clinical Trial Registration: ACTRN12621000567820

## Key Points

**Question:**

Can germicidal UV light (GUV) appliances in common spaces reduce the incidence of acute respiratory infections (ARIs) in long-term care facilities for older adults?

**Findings:**

In this randomized clinical trial that recorded 596 infections over 211 952 bed-days, GUV appliances did not result in a significant difference in the incidence rate per zone per cycle. When modeling ARIs across all cycles of the study, GUV appliances significantly reduced infections by 0.319 infections per week, equating to a 12.2% difference.

**Meaning:**

The trial findings suggest that GUV appliances did not reduce the incidence rate of ARIs within study cycles but did significantly reduce the total numbers of ARIs among older adult residents of long-term care facilities.

## Teaser

This randomized clinical trial examines whether germicidal UV appliances reduce acute respiratory infection incidence in long-term care facilities for older adults.

## Introduction

Outbreaks of common respiratory viruses, such as influenza, respiratory syncytial virus, and SARS-CoV-2, are associated with high rates of hospitalization and death for residents of long-term care facilities for older adults (LTCFs; also termed *residential aged care* or *nursing homes*).^1,2,3,4,5^ Infection control measures for common respiratory viruses focus on contact or droplet transmission. However, when airborne transmission occurs via infectious respiratory particles,^6^ standard precautions, like physical distancing, mask use, and hand hygiene, reduce, but do not eliminate, the risk of infection by viral aerosols.^7,8,9,10,11,12^ Increased rates of air exchange can reduce the risk of airborne viral transmission but are associated with considerable heating and cooling costs.^13^ Therefore, alternative strategies that can protect vulnerable older adult populations from seasonal respiratory virus outbreaks and future pandemics are urgently needed.

Germicidal UV (GUV) air sterilization appliances, also known as UV germicidal irradiation (UVGI) appliances, use UV light to kill airborne viral, bacterial, and fungal organisms as they pass through a disinfection zone as a consequence of passive or fan-assisted air circulation. GUV light appliances have been shown to be highly effective in killing airborne viral pathogens, including influenza,^14^ tuberculosis,^15^ SARS-CoV-1,^16^ and other human coronaviruses^17^ under laboratory conditions. While commercially available appliances have low associated running costs, can be used in parallel to existing infection control measures,^18,19^ and do not require changes in the practices of LTCF staff or residents,^20,21^ to our knowledge they are yet to be examined in health care settings. We report to our knowledge the first multicenter, pragmatic, cluster randomized clinical trial to evaluate the efficacy of commercially available GUV light appliances in reducing rates of airborne respiratory viral transmission in LTCFs.

## Methods

### Trial Design

**Figure 1.**
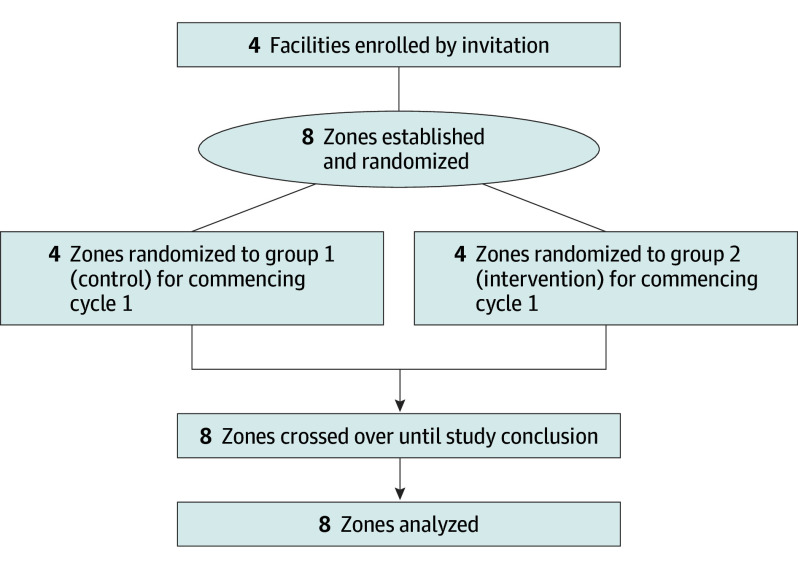
Study Flow Diagram Flow of long-term care facilities for older adults in South Australia targeted for the PETRA (Prevention of SARS-CoV-2 Transmission in Residential Aged Care Using UV Light) study, including the intervention, facilities enrolled, and facilities analyzed.

The Prevention of SARS-CoV-2 Transmission in Residential Aged Care Using UV Light (PETRA) study was a multicenter, pragmatic, cluster randomized clinical trial that used a 2-arm, double crossover, randomized, controlled design (Supplement 1 and Supplement 2).^22^ Trial approval was granted by the Bellberry Limited Ethics Committee. Clusters included matched discrete communal zones within each facility, including corridors, lobbies, and dining rooms. Resident rooms, amenities, and staff-only areas were excluded due to limited resident interaction in these spaces and concerns about disrupting residents’ private environments. Paired zones within clusters were randomly allocated to control or intervention in the initial cycle (1:1) (Figure 1), commencing on August 31, 2021. Facility staff and residents were not masked to control or intervention cycles.

Modification of the original trial design^23^ was necessitated by factors arising from the COVID-19 pandemic (detailed in the eMethods in Supplement 3). This included extending from 2 continuous assessment cycles to 7, encompassing 2 complete winter respiratory virus seasons (concluding on November 13, 2023). The study adhered to the Consolidated Standards of Reporting Trials Extension (CONSORT Extension) reporting guideline for cluster randomized trials.

### Participants

In Australia, long-term care is largely federally subsidized and delivered to approximately 1.5 million older people in LTCFs or at home by not-for-profit, for-profit, and government-operated services. While LTCFs can provide some short-term services, such as respite care, this represents a small proportion of the total resident cohort.

LTCFs in metropolitan and regional South Australia were recruited pragmatically if they could subdivide communal spaces into discrete areas (zones) to enable concurrent comparison of interventions in cohorts that were otherwise subject to the same infection control practices. Four LTCFs in South Australia participated in the cluster randomized trial (Figure 1), each providing 2 discrete matched zones (n = 8).

Some LTCFs included memory support units (MSUs) to provide specialist care for people living with behavioral and psychological symptoms of dementia. A specific subanalysis was performed to determine the efficacy of GUV appliance use within MSUs.

### Interventions

Retrofitting and use of commercially available GUV light appliances (LAF Technologies) was guided by qualified engineers and balanced equally across paired zones (eFigure 1 in Supplement 3). A combination of UV-FLOW-C wall-mounted and ceiling-mounted systems, UV-FAN M2/95HP, and UV-FAN-XS wall-mounted air purifiers were used (detailed in the eMethods in Supplement 3). Appliances were accredited by the National Association of Testing Authorities (ISO21501-4; ISO9001 quality accredited and ISO9001:2015 certified).

GUV appliances were switched off during control periods and run continuously during intervention periods. Control and intervention arms were run in parallel within each facility. Arm 1 (intervention) involved a 6-week GUV intervention period, followed by a 2-week washout, while arm 2 (control) involved a 6-week control period followed by a 2-week washout and crossover (eFigure 2 in Supplement 3).

### Outcomes

The primary outcome was the incidence rate ratio (IRR) of combined acute respiratory infections (ARIs), which were defined according to local and national health authorities^24,25^ and international guidelines.^26^ Residents met the primary outcome based on the clinical definition of symptomatic respiratory infection, which was sudden onset of symptoms, at least 1 of 3 respiratory symptoms (new or worsening cough, sore throat, and shortness of breath), and at least 1 of 4 systemic symptoms (fever or feverishness, headache, malaise, and myalgia). Case definition was met even when no swab test was performed, when the swab result was negative, and/or when individuals had positive test results during a diagnostic or screening test for respiratory infections, in accordance with national and local guidelines (eMethods in Supplement 3). Adverse events, concerns, harms, or unintended effects were actively monitored by facility staff and communicated to study personnel.

A priori secondary outcomes included rates of ARI-associated hospitalization, detection of respiratory viruses in air and surface samples, and virus genomic characterization. However, mandated COVID-19 infection control measures resulted in access to facilities being limited, preventing the collection of necessary samples. Considerable pressure on LTCF staff, hospitals, and diagnostic services also meant that access to hospitalization data was reduced, and genomic testing was prioritized for public health surveillance. Therefore, these secondary outcome measures were omitted.

### Sample Size

The trial sample size was based on the original protocol for a randomized, 4-period, double crossover control design in which each LTCF contained zones assigned to intervention and the control conditions during 2 consecutive respiratory infection seasons.^22^ The incidence rate of influenza infections was used to estimate power. A sample size of 8 zones, with a mean size of 40 residents per zone, was estimated to provide 89% power to detect a 50% reduction in the rate of symptomatic infections. This calculation assumed a mean of 35 days of follow-up per resident per 6-week period, a coefficient of variation for the zone event rate within each arm of 50%, an intraclass correlation within facilities of ρ = 0.03, a within-zone intraclass correlation of ρ = 0.20, a total of 4 measurement periods per zone (2 per season) and a variance inflation factor = (1 − ρ) / 4 = 0.2 for the relative number of zones required in total compared with a parallel group design.

### Randomization: Sequence Generation

Zones were paired within facilities and randomized to the intervention or control condition, respectively, for the first cycle (eMethods in Supplement 3). Zones were arranged to simplify the operational logistics of delivering an intervention that accommodated different building characteristics and layouts. This arrangement obviated the need for individual resident consent within the LTCF (Bellberry Limited Ethics Committee; 2021-04-403). The trial statistician was masked to intervention groups throughout the analysis.

### Statistical Methods

ARI incidence rates were calculated as the mean (95% CI) number of cases per zone per cycle and mean (95% CI) number of cases per 1000 bed-days. Differences in infection incidence rates were assessed using Poisson regression with mixed effects, with infection count as the dependent variable. The fixed effects were group (control and intervention) and cycle (1-7, categorical), and the random effect was facility zone (1-8, categorical). The logarithm of the exposure duration for each group (bed-days) was the offset term. Additional analyses included an intervention × cycle interaction term as a fixed effect. Differences between groups are reported as the mean difference (95% CI) in number of cases and IRR (95% CI).

An a priori sensitivity analysis was performed to adjust infection incidence to account for viral incubation, in which the incidence date was considered 3 days before ARI onset. Additionally, the effect of excluding residents within MSUs was assessed.

Due to the extended study duration, trends in the underlying rates of infections were assessed a posteriori using time-series regression, as detailed in the eMethods in Supplement 3. Statistical significance for all hypothesis testing was set using a 2-sided type 1 error rate of α = .05. A mixed-effects Poisson regression was performed in Stata (release 17; StataCorp) using the ‘mepossion’ command. The time-series analysis was performed in SAS (release 3.81; SAS Institute) using the PROC TIMESERIES and PROC AUTOREG procedures.

## Results

### Trial Participants and Baseline Characteristics

Four LTCFs completed the 110-week, 7-cycle study from August 31, 2021, to November 13, 2023 (Figure 1), including 3 metropolitan not-for-profit facilities and 1 rural public facility (Table 1). No facilities or zones withdrew, were lost to follow-up, or were excluded from analysis. No adverse events, harms, or unintended effects were reported.

**Table 1.** Characteristics of Enrolled Long-Term Care Facilities for Older Adults and Residents Who Acquired an Acute Respiratory Infection

Characteristic	No. (%)			
	Facility 1	Facility 2	Facility 3	Facility 4
Facility				
Organization type	Not for profit	Not for profit	Not for profit	Government
Services	Private	Private	Private	Public
Modified Monash Model location^a^	1	1	1	5
Beds available	115	75	110	80
Beds occupied, mean	108 (94)	73 (97)	106 (96)	69 (86)
GUV devices used, No.	47	34	61	37
Resident				
Acute respiratory infection events	261 (55)	76 (16)	92 (19)	46 (10)
Age, median (IQR), y	89 (82-93)	85 (80-90)	88 (83-94)	86 (80-91)
Sex				
Female	197 (75)	57 (75)	64 (70)	27 (59)
Male	64 (25)	19 (25)	28 (30)	19 (41)
Located in memory support area	75 (29)	0 (0)	19 (21)	5 (11)

Abbreviation: GUV, germicidal UV.

^a^ The Modified Monash Mode 2019 is a geographical classification that categorizes different areas in Australia into 7 remoteness categories across metropolitan, regional, rural, and remote areas according to geographical remoteness, as defined by the Australian Bureau of Statistics, and town size.

A total of 380 beds were available across all facilities, with a mean (SD) occupancy of 94% (0.05%) during the study period, representing 211 952 bed-days (Table 1). Facility infection control practices at study commencement aligned with local and national regulations (eTable 1 in Supplement 3). Changes to local, state, and national infection control policies during the study period are detailed in eFigure 3A in Supplement 3. The burden of acute respiratory infections within the wider South Australian population is presented in eFigure 3B in Supplement 3. These data were derived from the analysis of submitted clinical samples by the state public pathology service and aggregated by week.

A total of 596 ARIs were identified during the study period. Of these, 121 occurred during washout periods. The characteristics of the remaining 475 cases are presented in eTable 2 in Supplement 3. The incidence distribution across all facilities and facility-level changes to infection control practices are presented in eFigure 4 in Supplement 3.

### Effect of GUV Intervention on the Incidence Rate of ARIs

**Figure 2.**
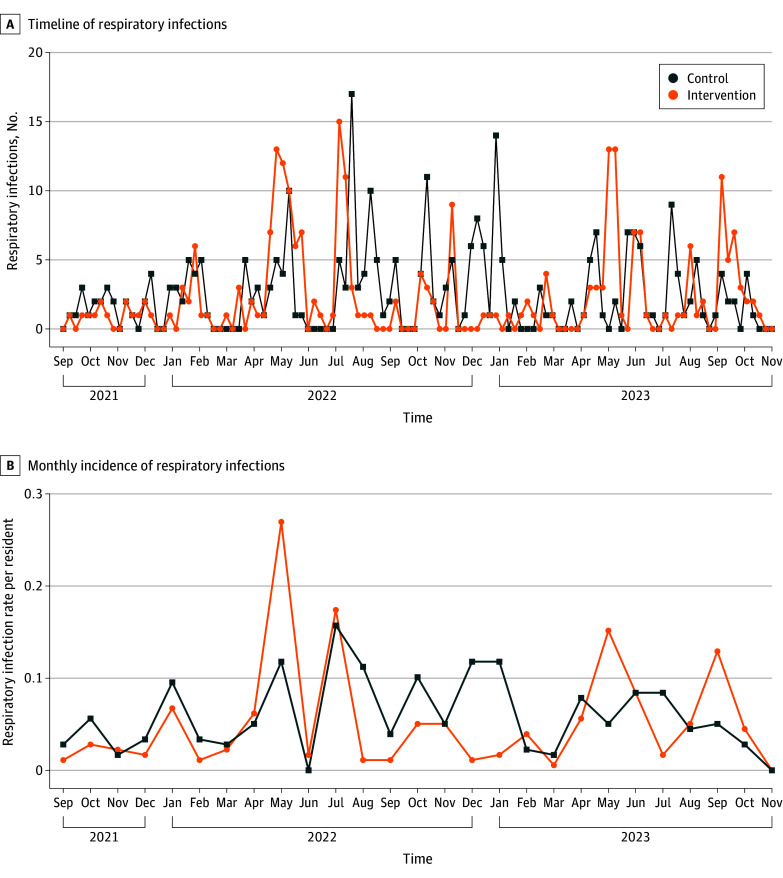
Incidence of Acute Respiratory Infections in PETRA (Prevention of SARS-CoV-2 Transmission in Residential Aged Care Using UV Light) A, The number and distribution of acute respiratory infection events across all long-term care facilities for older adults in PETRA from August 31, 2021, to November 13, 2023, among residents during periods of no intervention (control), or germicidal UV light (GUV) activity (intervention). B, The incidence rate of respiratory infections per resident per month in PETRA for control or intervention.

Two hundred and forty-eight events were reported in the control arm and 227 in the intervention arm (Figure 2). The mean number of estimated events in the control arm was 4.17 per zone per cycle (95% CI, 2.43-5.91), and 3.81 per zone per cycle (95% CI, 2.21-5.41) in the intervention arm (Table 2). This equated to 2.37 (95% CI, 1.69-3.05) infections per 1000 bed-days in the control arm and 2.17 (95% CI, 0.42-3.92) infections per 1000 bed-days in the intervention arm. The IRR was 0.91 (95% CI, 0.77-1.09; *P* = .33) and the overall difference in the number of infections per zone per cycle during the intervention was −0.36 (95% CI, −1.09 to 0.37).

**Table 2.** Incidence Rates of Respiratory Infections per Zone per Cycle in Control vs Intervention Conditions

Condition	Recorded events, No.	Mean (95% CI)		Incidence rate ratio (95% CI)^a^	Intervention vs control, estimated mean difference in infections per zone per cycle (95% CI)^b^	*P* value^a^
		Infections per zone per cycle	Infections per 1000 bed-days			
Control	248	4.17 (2.43 to 5.91)	2.37 (1.69 to 3.05)	1 [Reference]	−0.36 (−1.09 to 0.37)	.33
Intervention	227	3.81 (2.21 to 5.41)	2.17 (0.42 to 3.92)	0.91 (0.77 to 1.09)		
Control (excluding MSU)	197	3.36 (1.94 to 4.78)	1.88 (1.28 to 2.49)	1 [Reference]	−0.32 (−0.98 to 0.34)	.34
Intervention (excluding MSU)	179	3.04 (1.75 to 4.33)	1.71 (0.75 to 2.67)	0.91 (0.74 to 1.11)		

Abbreviation: MSU, memory support unit.

^a^ From mixed-effects Poisson regression model with zone as a random effect.

^b^ The estimated difference in number of infections per zone for intervention vs control conditions for each cycle of the study. The control condition is the reference. Also shown are the intervention effects after excluding events that occurred in MSUs.

After excluding cases in MSUs, 197 events were reported in the control arm and 179 in the intervention arm, with a mean number of estimated events per zone per cycle of 3.36 (95% CI, 1.94-4.78) and 3.04 (95% CI, 1.75-4.33), respectively. This equated to 1.88 (95% CI, 1.28-2.49) infections per 1000 bed-days in the control arm and 1.71 (95% CI, 0.75-2.67) infections per 1000 bed-days in the intervention arm and an IRR of 0.91 (95% CI, 0.74-1.11; *P* = .34) and overall difference in the number of infections per zone per cycle of −0.32 (95% CI, −0.98 to 0.34) (Table 2).

### Cumulative Incidence of Respiratory Infections in Response to the GUV Intervention

**Figure 3.**
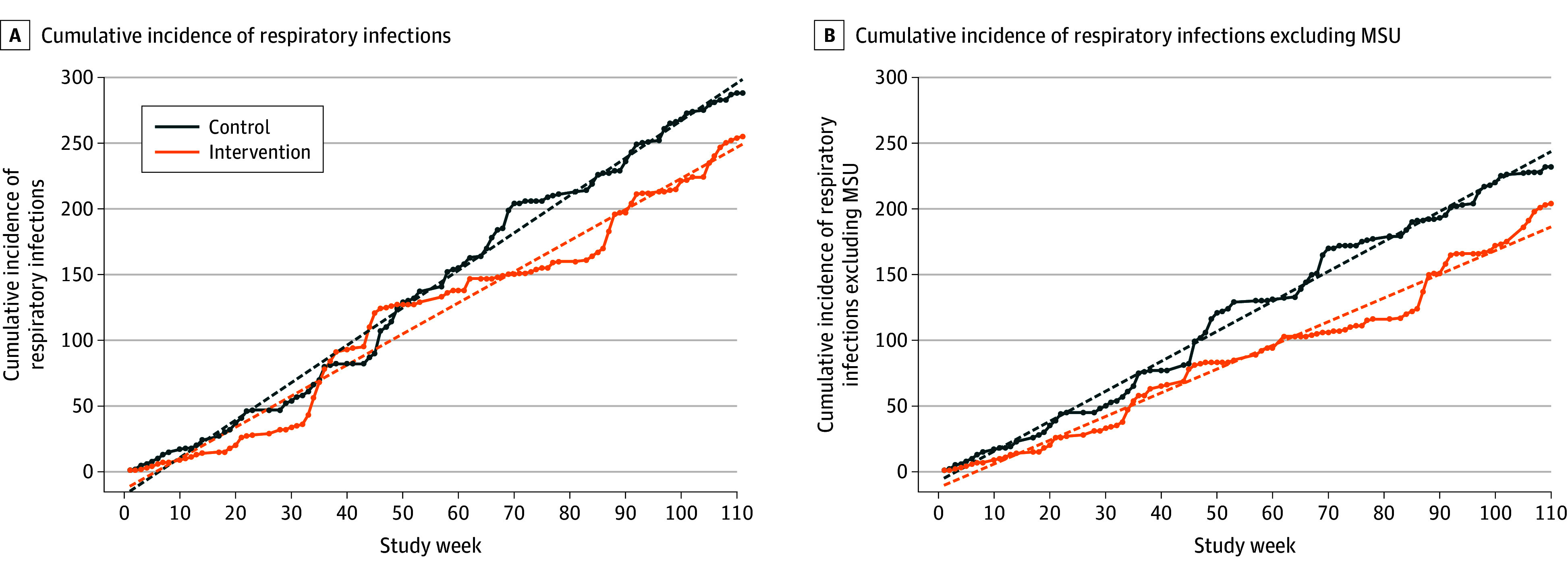
Cumulative Incidence of Acute Respiratory Infections in Control vs Intervention Conditions The observed (solid) and predicted (dashed) cumulative incidence of respiratory infections of the control and intervention conditions over 110 consecutive weeks, including (A) and excluding (B) events in memory support units (MSUs). Predicted series and trends were calculated with autoregressive modeling after removing level 2 autocorrelation.

A linear increase in the cumulative incidence of infections over time occurred within each condition. Observed and predicted cases, as well as predicted trends for the time-series analysis, are presented in Figure 3. Infections increased in the control arm at a rate of 2.61 (95% CI, 2.51-2.70) infections per week and the intervention arm at a rate of 2.29 (95% CI, 2.06-2.51) infections per week, equating to 0.32 (95% CI, 0.10-0.54; *P* = .004) fewer infections per week in the intervention arm or a 12.2% difference (Figure 3A; eTable 3 in Supplement 3).

After excluding events that occurred in MSUs, infections in the control arm increased at a rate of 2.14 (95% CI, 2.04-2.24) infections per week, while the intervention arm increased at a rate of 1.73 (95% CI, 1.54-1.92) infections per week, equating to 0.41 (95% CI, 0.21-0.60; *P* < .001) fewer infections per week in the intervention arm (Figure 3B; eTable 3 in Supplement 3).

### Sensitivity Analyses

Two sensitivity analyses were performed: (1) a 3-day offset to case inclusion dates to reflect viral incubation and (2) exclusion of MSU cases combined with a 3-day case inclusion offset (eTable 4 in Supplement 3). After adjusting for a 3-day offset, there was an overall IRR of 0.93 (95% CI, 0.78-1.11; *P* = .43), with an overall difference in the number of infections per zone per cycle during intervention of −0.29 (95% CI, −1.01 to 0.44). After the combined MSU case exclusion and adjusted window, there was an overall IRR of 0.95 (95% CI, 0.77-1.16; *P* = .58), with an overall difference in the number of infections per zone per cycle during intervention of −0.18 (95% CI, −0.82 to 0.47). IRRs were consistent between zones, with the exception of facility 3, where the IRR was significantly higher in both zones (eTable 5 in Supplement 3).

The differences in cumulative incidence and estimated infection rate within the control and intervention conditions remained after sensitivity analyses (eFigure 5 and eTable 6 in Supplement 3). After adjusting for a 3-day offset, the intervention arm was associated with 0.279 fewer infections per week (SE, 0.107; *P* = .01) compared with the control group. Combined MSU case exclusion and a 3-day offset resulted in 0.319 fewer infections per week (SE, 0.104; *P* < .002) compared with the control group. There were 13 instances of resident hospitalization (public hospitals only) during the control arm, compared with 9 during the intervention arm (eTable 7 in Supplement 3).

## Discussion

The COVID-19 pandemic resulted in renewed focus on infection control and prevention practices, including the need for effective strategies to reduce airborne transmission, particularly in LTCFs.^27^ GUV appliances can be used with minimal disruption and represent a potential adjunct to existing infection control measures. However, despite growing interest in this technology, a recent systematic review highlighted limited clinical evidence.^28^

We report the use of GUV appliances in LTCF communal areas to result in a nonsignificant decrease in ARI incidence rates per zone per cycle (the primary study outcome measure). However, time-series modeling performed on the extended assessment period (28 to 110 weeks) showed a statistically significant 12.2% reduction in weekly ARIs (0.319 fewer per week). This difference in findings likely reflects the random timing of infections, variations in infection rates between cycles, and external environmental factors and highlights the importance of considering not only the IRR at a specific end point but also the underlying long-term trends in incidence rates for each condition.

Our study estimated the causal effect of the intervention to be an approximately 9% reduction in infections. When applied to the ARI rate in the control arm, such a reduction equates to 92 fewer ARIs per 1000 residents annually. While falling short of the 20% benchmark that is often considered a clinically meaningful change for an individual, such a reduction could translate to a very meaningful effect from a public health perspective, for which the aggregate benefit of even small individual improvements becomes substantial.^29^ This potential was highlighted by the rate of hospitalization associated with ARIs being 3 to 9 times higher^30^ and the mortality rate being 9 to 11 times higher in populations of older adults.^31,32^ Moreover, the effect of GUV appliances within LTCFs might be further augmented through a refined strategy for retrofitting and use or the integration of GUV technology into ducted heating, ventilation, and air conditioning systems to provide more comprehensive air sterilization.

Standard infection control measures, such as mask-wearing and physical distancing, are often impractical within MSUs, resulting in higher infection transmission rates. Exclusion of MSU residents resulted in a reduction in the total number of events and a decrease in the number of infections per zone per cycle in both arms but no changes in the IRRs. However, the reduction in ARIs resulted in a statistically significant difference in cumulative incidence slopes between the intervention and control arms. This change in cumulative incidence was notable between weeks 30 and 50 of the trial and corresponded to confirmed respiratory infection outbreaks within participating MSUs.

ARIs resulting from transmission events during a nonintervention period may become symptomatic or detectable early in the assessment period, while transmission events at the end of assessment periods may only be identified during washout.^33^ Therefore, we assessed the potential effect of viral incubation periods on the observed effects of the intervention. Accounting for viral incubation by applying a 3-day offset had only a modest effect, with a narrowing of 2% in the mean IRR.

### Limitations

Our study had limitations. First, due to the unprecedented nature of the COVID-19 pandemic and ongoing changes in associated public health measures, data to inform power calculations during the study design were not available. Consequently, in response to unexpectedly low ARI rates during the initial study period, it was necessary to increase the number of assessment cycles. Second, the mandated limitation of LTCF access to essential workers meant that secondary outcomes that required collection of environmental samples could not be pursued. Third, the use of deidentified data in relation to ARI incidence meant that events had to be considered independent. Fourth, the movement of residents and staff outside of intervention zones was unrestricted, and the potential for pathogen transmission between intervention and control areas could not be excluded. Fifth, extreme pressure on diagnostic services during the pandemic meant that it was not possible to confirm the cause of symptomatic ARIs in all cases. Several respiratory samples from facility residents underwent targeted SARS-CoV-2 screening only. Consequently, many symptomatic ARIs were not confirmed through laboratory testing as assays for potential causative agents were not performed, with no opportunity to undertake retrospective testing due to destructive sample processing. Finally, our assessment was based on commercially available GUV appliances, and further studies are required to understand how the type and deployment pattern of GUV appliances influences their effect.

Despite these limitations and the changing effect of the COVID-19 pandemic, this trial demonstrated the effectiveness of an adjunct infection control strategy to address airborne pathogen transmission in a health care setting. This highlighted the potential of GUV-based strategies, if shown to be cost-effective, in preventing seasonal respiratory infections and protecting vulnerable populations against future outbreaks of novel viral pathogens.

## Conclusions

While this randomized clinical trial found that use of GUV appliances did not reduce the ARI incidence rate within study cycles, it did reduce the total numbers of ARIs by the study conclusion. GUV-based strategies are a potential adjunct to existing infection control practices for vulnerable residential populations.

## Notes

Supplement 1. Trial protocol

Supplement 2. Statistical analysis plan

Supplement 3. eMethods. Trial design, participants, interventions, outcomes, randomisation, statistical methods, and statistical software eFigure 1. Example deployment of GUV appliances across a LTCF zone eFigure 2. PETRA trial stages eFigure 3. Respiratory virus circulation in South Australia (SA) corresponding with changes to national and state-wide changes in infection control practices eTable 1. Baseline infection control practices of enrolled long-term aged care facilities at commencement of PETRA eTable 2. Characteristics of residents with reported acute respiratory infection events by facility during the control or intervention periods eFigure 4. Incidence of respiratory infections in PETRA in response to changes in facility infection control practices eTable 3. Cumulative incidence of respiratory infections in control versus intervention conditions eTable 4. Number of infections and incidence rate ratio (IRR) for acute respiratory infections after sensitivity adjustments eTable 5. Number of infections and incidence rate ratio (IRR) for acute respiratory infections by zone after sensitivity adjustments eFigure 5. Cumulative incidence of respiratory infections in control versus intervention after sensitivity adjustments and exclusions eTable 6. Estimated increase in infections per week for residents in intervention versus control groups after sensitivity adjustments and exclusions eTable 7. Incidence of public hospitalisation for complications associated with respiratory infection in intervention versus control groups

Supplement 4. Data sharing statement
