# Supplementary material for: Germicidal UV Light and Incidence of Acute Respiratory Infection in Long-Term Care for Older Adults: A Randomized Clinical Trial
Source: JAMA Intern Med. 2025 Jul 28;185(9):1128–35. doi: 10.1001/jamainternmed.2025.3388 (PMC12305439; doi:10.1001/jamainternmed.2025.3388)
Supplement: Supplement 1. — Trial protocol [file jamainternmed-e253388-s001.pdf]

Study Protocol

The PETRA Study:

Prevention of SARS-CoV-2 (COVID-19) Transmission in Residential Aged Care using ultraviolet light (PETRA): a parallel crossover randomised controlled trial

Project Title:

Prevention of SARS-CoV-2 (COVID-19) Transmission in Residential Aged Care using ultraviolet light (PETRA): a parallel crossover randomised controlled trial study protocol

Principal Investigator: Professor Geraint Rogers

Contents

1. Study Registration: 3
2. Key Study Investigators: 3
3. Resources: 4
4. Site Location 4
5. Purpose of the Study 4
6. Background Information: 4
7. Hypothesis: 8
8. Methodology and Procedures: 8
9. Site Selection: 12
10. Waiver of Consent: 13
11. Data Management: 17
12. Timeframes, Outcomes and Future Plans: 18
13. Risk and Monitoring 19
14. Study Governance: 26
15. References: 27

1. Study Registration:

Australian and New Zealand Clinical Trial Registration number ACTRN12621000567820.

2. Key Study Investigators:

Position: **Principal Investigator**	
Name: Professor Geraint Rogers	
Institutional affiliation: South Australian Health & Medical Research Institute; Flinders University; Flinders Medical Centre	
Department and department address: Infection & Immunity, SAHMRI, Flinders Dr, Bedford Park SA 5042	
Contact details:<br>☒ I am the contact person for this project	Phone: +61 0403558251<br>Email: geraint.rogers@flinders.edu.au

Position: **Trial Statistician**	
Name: Professor Richard Woodman	
Institutional affiliation: Flinders Health and Medical Research Centre, Flinders University; South Australian Health and Medical Research Institute	
Department and department address: Flinders University, Health Sciences Building, Flinders Dr, Bedford Park SA 5042	
Contact details:<br>☐ I am the contact person for this project	Phone: +61 8 7221 8537<br>Email: richard.woodman@flinders.edu.au

Position: **Study Manager**	
Name: Dr Amanda Brass	
Institutional affiliation: South Australian Health & Medical Research Institute	
Department and department address: Infection & Immunity, SAHMRI, Flinders Dr, Bedford Park SA 5042	
Contact details:<br>☐ I am the contact person for this project	Phone: +61 8 8128 4263<br>Email: Amanda.Brass@sahmri.com

3. Resources:

**Resources:** All research staff, research assets (e.g. lab/IT equipment) and financial support are in place for the study. The study team is working closely with residential aged care facilities within South Australia and has secured study partnerships and sites.
**Funding support:** Funding for this study, to the value of AUD$1,366,094, has been secured from the National Health and Medical Research Council's Medical Research Future Fund (GNT2005904).
**Insurance details:** This study has been listed on the SAHMRI clinical trial register and as such, all SAHMRI staff working on this project are covered by SAHMRI insurance. In addition, the Germicidal Ultraviolet (GUV) devices used in this study have been noted under the SAHMRI public & products liability and clinical trial policy.

4. Site Location

**Administrative site:** All research and management of the study will be based at SAHMRI, Flinders Medical Centre, Bedford Park SA 5042 (08 8128 4263).
**Intervention site:** The GUV intervention will be deployed in four undisclosed residential aged care facilities across metropolitan and regional South Australia.

5. Purpose of the Study

Residential aged care facilities (RACF) have experienced catastrophic outbreaks of COVID-19. Rapid transmission of SARS-CoV-2 within facilities, combined with the increased likelihood of severe illness or death due to resident age, comorbidities and frailty, resulted in the highest mortality rate of any population (29.5%). Eighty-three separate outbreaks have occurred in Australian residential aged care facilities, despite increasing efforts to prevent SARS-CoV-2 transmission, including by limiting facility access, preventing staff from working at multiple sites, using personal protective equipment, increasing hand hygiene and environmental cleaning, and implementing social distancing. It is critically important that more effective strategies to protect those in aged care from COVID-19 are identified rapidly.

Given the scale of the threat posed by COVID-19 to aged care residents, it is imperative that the potential for airborne transmission in these settings is mitigated. The Prevention of COVID-19 Transmission in Residential Aged Care using ultraviolet light (PETRA) study will determine the impact of rapidly deployable, cost-effective measures to reduce airborne viral transmission in RACF settings. These interventions will be assessed in a world-first multicentre randomised cluster-controlled trial of air-sterilisation strategies.

6. Background Information:

Literature Review: Despite SARS-CoV-2 (COVID-19) representing a greater threat to aged care residents than to any other population, the development of RACF-specific protective measures has been largely neglected. Domestically and internationally, more than 99% of registered COVID-19 trials focus on vaccine development or pharmaceutical interventions without considering aged or long-term care populations[1,2].

The lack of effective preventative measures for aged care settings, combined with the

high morbidity and mortality of COVID-19 in this age group, has proven calamitous[3]. The speed at which COVID-19 can spread within RACF settings is highlighted by the results of widespread testing in Belgian RACF, which revealed 73% of employees and 69% of residents who tested positive for COVID-19 to be asymptomatic at the time of testing[4]. Such rapid transmission can overwhelm infection control responses[3], and it is essential that transmission prevention strategies are designed and tailored specifically for these settings.

The involvement of airborne transmission to overall COVID-19 epidemiology is an area of ongoing debate[5]. An increasing body of evidence supports bioaerosol-mediated spread[6,7], however, the overall contribution of this route to outbreaks remains unclear. Understanding of the principal routes through which COVID-19 is transmitted remains poor. Recent reports suggests that large airborne droplets, which travel relatively short distances before settling on surfaces, and smaller airborne droplet nuclei (bioaerosols), which can remain suspended in the air for considerably longer, both contribute to transmission[6,7]. An airborne component of COVID-19 transmission would be consistent with other respiratory viruses, such as SARS-CoV-1, Middle Eastern respiratory syndrome coronavirus (MERS-CoV), and influenza[8,9]. At present, current infection control measures (e.g. wearing of surgical masks, handwashing, environmental cleaning) focus almost exclusively on droplet-based and fomite-related transmission routes.

A range of measures are used in other clinical and non-clinical contexts to prevent airborne transmission of viral and bacterial pathogens, which also have considerable potential to reduce COVID-19 transmission in RACFs. These approaches could be applied in parallel to existing infection prevention measures, and implemented in a rapid, cost-effective, and non-disruptive manner. Importantly, they do not rely on changes in the behaviour or practices of residents or staff (as required for safe use of Personal Protective Equipment (PPE), for example). These measures include:

Reduced air recirculation: Recirculation of air is common practice in aged care facilities due to the associated reduction in heating and cooling costs. However, it also increases exposure to airborne particles, including viruses[10]. Modifying heating, ventilation and air conditioning (HVAC) systems to increase air exchange can therefore reduce the risk of viral transmission[10].

Germicidal ultraviolet (GUV): GUV is highly effective in killing a range of viral pathogens, including airborne influenza, and SARS-CoV-1[11,12], as well as other human coronaviruses, such as alpha HCoV-229E and beta HCoV-OC43[13]. Upper-room GUV systems direct GUV light into the upper air zone and exposing air as it circulates through natural convection. GUV systems can also be placed within HVAC ducts, or enclosed within fan units that draw air into a germicidal compartment.

Air filtration: High-efficiency particulate air (HEPA) filtration is not typical within standard air conditioning systems. However, HEPA filtration can be effective in reducing airborne viral circulation[14], particularly in conjunction with GUV.

Of the available approaches, GUV appliances have the greatest potential. Commercially available upper-room GUV (with effective air-mixing) has been shown to reduce airborne tuberculosis transmission at a rate equivalent to adding 24 room air changes per hour[15]. and its high efficacy against airborne viruses, including influenza and SARS-CoV-1, has been clearly demonstrated[11,12]. Similarly, when GUV has been deployed in standalone fan-based systems, it has been shown to result in significant reductions in both viral viability and rates of symptomatic respiratory infections[16]. These GUV modalities might therefore be combined to achieve effective air disinfection in accordance with RACF

layout, occupancy, and air-flow setups.

Investigational Product: Commercially available germicidal ultraviolet (GUV) appliances: UV-FLOW-C wall-mounted system, UV-FAN M2/95HP air purification device, UV-FAN-XS wall-mounted air purifier; LAF Technologies, Melbourne, Australia. The devices are NATA accredited (ISO21501-4), Eco Warranty accredited, ISO9001 quality accredited, and ISO9001:2015 certified.

Summary of previous findings: Commercially available upper-room GUV (with effective air-mixing) has been shown to reduce airborne tuberculosis transmission at a rate equivalent to adding 24 room air changes per hour[15], and its high efficacy against airborne viruses, including influenza and SARS-CoV-1, has been clearly demonstrated[11,12]. Similarly, when GUV has been deployed as standalone fan-based systems, it has been shown to result in significant reductions in both viral viability and rates of symptomatic respiratory infections[16]. These GUV modalities could therefore be combined to achieve effective air disinfection in accordance with RACF layout, occupancy, and air-flow setups.

Summary of risks and benefits: Despite clear potential for airborne transmission to contribute to SARS-CoV-2 outbreaks in RACF settings[17], current strategies to protect more than 210,000 Australians currently in residential aged care do not include any measures to address this specific threat. By assessing a potentially transformative infection control strategy, our study addresses a critically important public health need. Reduction of the risk of SARS-CoV-2 transmission, even modestly, could prevent considerable loss of life. In addition to the threat of COVID-19, this study will address the considerable burden or morbidity and mortality associated with other respiratory viral pathogens (e.g. RSV, influenza) and non-respiratory viruses (e.g. enterovirus and norovirus) in aged care settings[18]. Each of these pathogens is transmissible via bioaerosols[19,20]. The threat that they pose to aged care residents, which has never been reduced to an acceptable level, will continue long after the COVID-19 pandemic has resolved. Indeed, it is notable that both overall mortality and confirmed influenza mortality in aged care to 31 July was higher in 2019 (n=33,383; 837) than overall and combined COVID/influenza mortality in 2020 (n=32,398; 580/28)[21]. Collateral reductions in outbreaks of infections caused by such pathogens would represent a considerable advance in the health and well-being of aged care residents, and ensure this setting was well prepared for future respiratory outbreaks or even pandemics.

The potential risk concerns direct exposure to UV-C light. This will be mitigated by installation of the units by certified contractors that will adhere to the installation instructions provided by the supplier. Contractors and maintenance workers will be provided with adequate training and instructions adhering to safety requirements when working with the devices. This may require wearing UV-C eye and skin protection when installing and ascending into the range of UV-C light exposure.

UV-C light will be completely guarded for both the wall-mounted UV-FAN-XS and portable/wall-mounted UV-FAN M2/95HP. The UV-FLOW-C device does emit UV-C light and is not shielded, but will only be installed at a minimum height of 2.2 metres. These devices will direct the UV-C light upwards and the range of spread will not drop below 2.2 metres, and therefore not within reach of persons present. The UV-FLOW will also have a dedicated circuit with a master switch that will power off units from a distance. All UV-C emissions will be measured (using a UVC254 light meter) following installation and at the start of the intervention period, to check for functionality and UV-C spread. The UV-C light emitted will have a peak emission of 254 nanometres and does not produce ozone. Warning signage will be on all UV-C devices.

Description and justification for intervention administration: The characteristics of aged care facilities vary, including the age of the building, facility layout, occupancy, rates of air change, existing infection control measures, staff procedures, and HVAC systems. Residents commonly dine together in shared areas, and also congregate for group recreational activities. Otherwise, it is typical for residents to spend a substantial portion of the time within their living quarters. Opportunities for airborne pathogen transmission are therefore particularly great during mealtimes, when residents come together for recreation, and when bioaerosols, generated within resident rooms, migrate into shared corridor and connective spaces. These exposures are compounded by air recirculation. The larger spaces used for meals, for example, tend to employ ducted HVAC systems with high levels of recirculation, with air intake and return units acting to distribute airborne particles between those present.

GUV is highly effective in killing a range of viral pathogens, including airborne influenza, and SARS-CoV-1[11,12], as well as other human coronaviruses, such as alpha HCoV-229E and beta HCoV-OC43[13]. Upper-room GUV systems direct GUV light into the upper air zone and exposing air as it circulates through natural convection. GUV systems can also be enclosed within fan units that draw air into a germicidal compartment. Facility permitting, our intervention will aim to involve a combined use of GUV approaches: ceiling- and wall-mounted units, situated in a pattern that provides air purification of connecting corridors and spaces between resident rooms, with additional units providing coverage to high traffic and shared spaces, such as lift areas and dining areas; and standalone fan-driven units used in occasional areas, such as cinema units, chapels, and treatment rooms (employed during and following area use). It is important to highlight that such mobile units also have considerable utility during facility outbreaks of pathogens, such as influenza and norovirus, where it is not possible to confine all residents to rooms in order to reduce interaction and the potential for transmission. This is the case, for example, in specialist sections for those with significant cognitive or memory impairment. *In situ* appliance performance will be tested according to ASHRAE Standard 185.1,30 and deployed in such a way as to achieve an equivalent level of coverage between comparator sites[22]. No GUV units will be installed in residents' bedrooms or private areas.

The intervention will occur for multiple cycles and overlap with historical peak influenza seasons, and include a treatment and a control arm:

- Arm 1 (treatment): the GUV intervention will be applied for a 6-week period, followed by a 2-week washout period (to account for respiratory virus incubation periods), and then a crossover to the control period for 6-weeks, followed by a final 2-week washout.
- Arm 2 (control): the control period will be applied for 6-weeks, followed by a 2-week washout period, and then a crossover to the GUV intervention will apply for 6-weeks, followed by a final 2-week washout.

Trial statement: The PETRA trial will be documented in compliance with this study protocol, Good Clinical Practice in Australia, and regulatory requirements.

Study population: Of Australian states and territories, South Australia (SA) is second only to Tasmania in the proportion of individuals that are aged over 65 years[23], and has the highest number of aged care residents in proportion to its population[24]. Our study will be undertaken in partnership with multiple aged care providers in SA, encompassing metropolitan and rural settings.
The study will be undertaken in undisclosed RACFs across multiple providers and locations. Collectively, they are broadly representative of RACFs more widely, in both

capacity and occupancy. The study settings have been selected based primarily on the ability to sub-divide resident populations into discrete areas that enable concurrent comparison of interventions in facility cohorts which are otherwise subject to the same facility practices (e.g. environmental cleaning, staffing, and social distancing). In many settings, such measures have already been implemented in response to the COVID-19 pandemic.

7. Hypothesis:

Hypothesis: We hypothesise that facility-tailored GUV strategies, as an adjunct to existing infection control measures, can substantially reduce rates of acute respiratory infection in RACF residents.

8. Methodology and Procedures:

Study Participants and Trial Period: The study will be undertaken in four residential aged care facilities across three providers, offering a total of approximately 400 occupied beds. Collectively, they are broadly representative of RACF more widely, in both capacity and occupancy. The study settings have been selected based primarily on the ability to sub-divide resident populations into discrete areas that enable concurrent comparison of interventions in facility cohorts which are otherwise subject to the same facility practices (e.g. environmental cleaning, staffing, and social distancing). In many settings, such measures have already been implemented in response to the COVID-19 pandemic.

The intervention phases of the study will be conducted from winter 2021 through to late 2023, including periods which overlap with the highest incidence of respiratory infection in residential aged care (**Figure 1**).

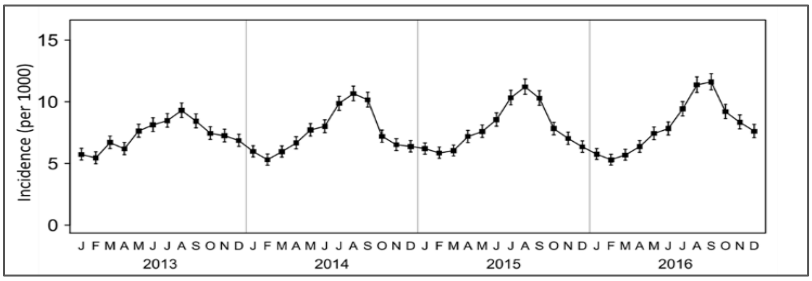

Figure 1: Hospitalisation for lower respiratory infection, sex and age-standardised (ROSA).

Trial Instrumentation and Design: The study is a parallel cross-over randomised controlled trial targeting air-sterilisation strategies to reduce the burden of airborne respiratory infections in residential aged care settings. The intervention will use commercially available upper-room germicidal ultraviolet (GUV) devices. We will compare adjunct air sterilisation measures to existing infection control measures. To account for differences in facility characteristics, assessment will be based on within-facility comparisons of discrete zones, using a parallel cross-over randomised controlled trial design. Where possible, paired zones will be highly similar in size, layout, and occupancy, allowing randomisation (using concealed random allocation

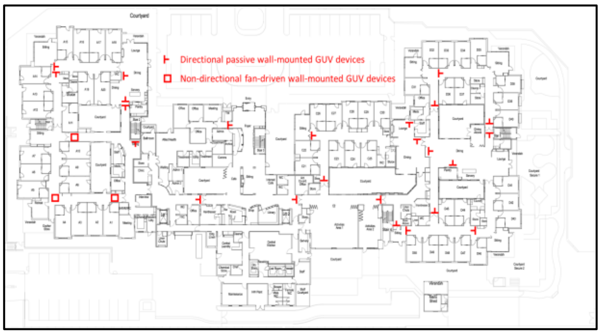

Figure 2: Example deployment of GUV devices within a RACF.

from a computer-generated random numbers table) to starting control/intervention order within the cross-over study design. Similar GUV strategies will be employed in paired areas to account for temporal variation in seasonal virus levels (the layout shown in **Figure 2**, for example, is identical to that of the floor above, with which it will be paired). Each pair will be randomised to begin the study schedule with either the intervention or the control period (i.e. one zone in each pair to start with the control period and the intervention, respectively). A cycle will consist of a six-week intervention period followed by a two-week washout period (to account for respiratory virus incubation periods), before crossover to the second intervention period (**Figure 3**). Each cycle will be continually repeated through to late 2023, including a minimum of a two-week washout period between each cycle. The trial is an open trial, with masking or blinding not used. Air purification will be continuous during the intervention periods. Any changes in existing infection control practices will be recorded. The study interventions will cycle through and overlap two winter seasons. This approach will maximise opportunities for rapid implementation of protective measures, as well as supporting the production of highly robust data.

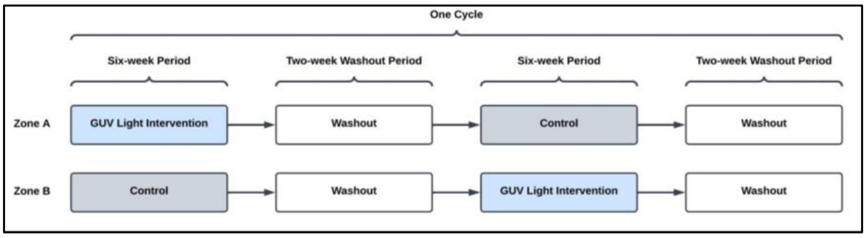

Figure 3: Study schedule

Each facility contains zones assigned to both the intervention and the control condition for each cycle (once for each condition in each of the study cycles). The intervention will include a treatment and a control/comparator arm:

- Arm 1 (intervention): The GUV intervention will be applied for a 6-week period, followed by a 2-week washout period (to account for respiratory virus incubation periods), and then a crossover to the control period for 6-weeks, followed by a final 2-week washout.
- Arm 2 (control): The control period will be applied for 6-weeks, followed by a 2-week washout period, and then a crossover to the GUV intervention will apply for 6-weeks, followed by a final 2-week washout.

Discontinuation Criteria & Accountability Procedures: RACFs are able to discontinue the study at any point in time. The study team will only discontinue the study if data is not supplied by the RACF, access is not granted or if there are safety issues. In the event that data from Year 1 has shown a significant benefit in reducing acute respiratory infections in RACFs, the continuation into Year 2 will be discussed, and reviewed with regards to continuing with Arm 2 (control - no GUV intervention).

Study Procedures: The characteristics of aged care facilities vary, including in their layout, occupancy, rates of air change, existing infection control measures, staff procedures, and HVAC systems. Residents commonly dine together in shared areas, and also congregate for group recreational activities. Otherwise, it is typical for residents to spend a substantial portion of the time within their living quarters. Opportunities for airborne pathogen transmission are therefore particularly great during meal-times, when residents come together for recreation, and when bioaerosols, generated within resident rooms, migrate into shared corridor and connective spaces. These exposures are compounded by air recirculation. Where possible, our intervention will involve the combined use of GUV approaches: ceiling- and wall-mounted units, situated in a pattern that provides air purification of connecting corridors and spaces between resident rooms, with additional

units providing coverage to high traffic and shared spaces, such as lift areas and dining areas; and standalone fan-driven units used in occasional areas, such as cinema units, chapels, and other function rooms. This approach will be achieved using commercially available appliances (**Figure 4**) including: ceiling- and wall-mounted (UV-FLOW-C, UV-FAN M2/95HP, and UV-FAN-XS, and standalone mobile UV-FAN M2/95HP; LAF Technologies, Melbourne).

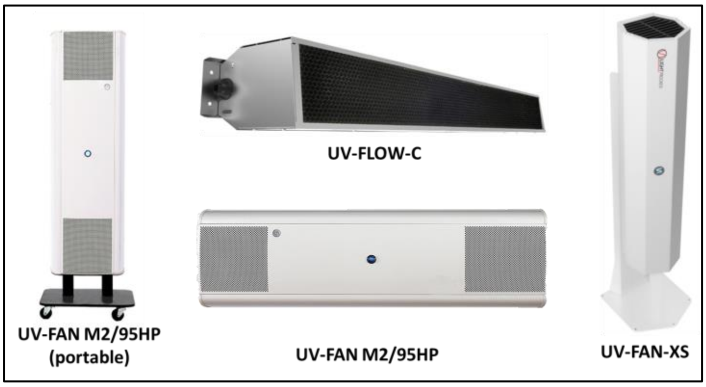

Figure 4: GUV appliances utilised.

An example deployment pattern is shown in **Figure 2**. This overview does not include fan-driven units for occasional rooms, which will be used for two such areas within each facility, selected based on regularity of use, and typical occupancy. It is important to highlight that such mobile units also have considerable utility during facility outbreaks of pathogens, such as influenza and norovirus, where it is not possible to confine all residents to rooms in order to reduce interaction and the potential for transmission. This is the case, for example, in specialist sections for those with significant cognitive or memory impairment. *In situ* appliance performance will be tested according to ASHRAE Standard 185.1,30 and deployed in such a way as to achieve an equivalent level of coverage between comparator sites.

Consent: The participation of residential aged care facilities in the study has been at the discretion of the provider CEOs and senior management, who act in the best interest of the residents. At any stage during the study, facilities will have the option to withdraw from the trial. Recruitment will not occur at the individual level (see Waiver of Consent). Residents will be communicated with by the study team and RACF staff regarding the trial to inform themselves and their families of the trial. All research data will be stored securely at SAHMRI in either a locked office or password protected in a REDCap database stored on the SAHMRI server. All study data will only be accessible to study staff.

Outcomes:

Primary outcome: Rate of symptomatic respiratory infection in residents of aged care facilities will be continuously recorded throughout the entirety of the study. The study will utilise the existing framework for surveillance of acute respiratory infection (ARI), including COVID-19 and influenza, in residential aged care facilities, the guidelines of which have been published by both national (communicable diseases network Australia) and local authorities (communicable diseases control branch of South Australia)[25]. They define acute respiratory infection clinically as the recent onset of new or worsening: cough, breathing difficulty, sore throat, nasal congestion, or runny nose. If a resident meets this definition, discussions with the treating GP will occur for testing by nucleic acid amplification using a nose and/or throat swab.

To ensure thorough and consistent case identification according to study definitions, the study manger will liaise directly with facility staff for the duration of the study to capture this information. During the intervention periods, respiratory infections will be counted where residents meet the clinical definition of an ARI and/or test positive for a respiratory virus through routine screening or diagnostic testing. The RACF clinical team representative will

follow the facility's standard surveillance program. Participating facilities will request medical officers send specimens to South Australian pathology laboratories where specimens sent for influenza testing are also routinely tested for a range of other respiratory pathogens including COVID-19 (SARS-CoV-2*),* influenza A, influenza B, parainfluenza virus 1, 2 and 3, adenovirus, rhinovirus, human metapneumovirus, respiratory syncytial virus, *Bordetella pertussis*, and *Mycoplasma pneumoniae*. Residents will have met the primary outcome based on fulfilling the symptomatic respiratory infection clinical definition, even if no swab was performed or if the swab result is negative, and/or if they test positive during a diagnostic or screening test for respiratory infections. All results of respiratory virus tests will be recorded and linked in a de-identified manner to the respective ARI event.

<u>Secondary outcome 1:</u> Rates of hospitalisation for complications associated with respiratory infection. Hospital admissions, or presentations at hospital emergency departments for complications associated with acute respiratory infection, will be recorded through facility notes where possible, including viral diagnostics and recorded on the study data capture sheet. The study manger/coordinator will liaise directly with facility staff for the duration of the study to capture this information, all data supplied to the research study team will be de-identified.

<u>Secondary outcome 2:</u> Respiratory virus detection and quantification in facility air samples. In addition to assessing respiratory infections in residents, levels of airborne and surface viral particles within the RACFs will be measured. The methodology used is not designed to distinguish between viable and non-viable viral particles, and these environmental assessments are not intended as a measure of infection risk. Rather, they provide a snapshot of the level of detectable viruses in the environment that could be missed from only testing symptomatic residents (i.e. as generated by asymptomatic staff, visitors or residents) – viral shedding by asymptomatic individuals has been described in relation to both common respiratory viruses[26] and COVID-19[27]. As an indirect measure of viral particle load, this study component will provide an additional basis to assess intervention impact.

Assessment of airborne respiratory virus carriage will be performed based on weekly air sample collection for the duration of the intervention periods (Coriolis micro liquid output air sampler; Bertin Instruments, Fortitude Valley, Queensland)[28, 29]. Levels of viral particles in air will be determined using quantitative, non-multiplexed versions of PCR assays.

<u>Secondary outcome 3:</u> Rate of respiratory viral detection and quantification in facility fomite samples. Samples will be collected weekly for the duration of the intervention periods from non-absorbent fomites with pre-wetted (0.25 strength Ringer's solution) polyester-tipped swabs, using a fixed area template[30]. Levels of viral particles on fomite samples will be determined using quantitative, non-multiplexed versions of PCR assays.

<u>Secondary outcome 4:</u> Rate of laboratory confirmed respiratory illnesses and genomic characteristics. Data will be captured on specimens sent to SA Pathology for respiratory virus testing during the study period. Rates will be calculated for individual respiratory viruses previously described. Viral nucleic acid will be extracted and stored by SA Pathology for all positive specimens. Viral sequencing will be undertaken to determine epidemiological links.

Additional measures: In addition to the study outcomes above, a number of other variables will be recorded. This will include RACF lockdowns in response to outbreaks of notifiable infections, or infections associated in rapid spread or high infectivity. Any such infection-related changes in facility operation will be recorded. In addition, mortality, cause of death,

and whether residents were receiving palliative care, may be determined via facility records.<br><br>**Statistical Justification:** Based on a randomised 4-period parallel cross-over control design in which each RACF contains zones assigned to both the intervention and the control condition twice (once for each condition in each of two consecutive respiratory infection seasons), we calculate that a sample size of n=8 zones (across four facilities, from three aged care providers), with an average size of n=40 residents per zone, will provide 89% power to detect a 50% reduction in rate of symptomatic infections i.e. five per 1000 person-days in the intervention group versus 10 per 1000 person-days in the control group. This calculation assumes an average of 35 days of follow-up for each resident for each of the six-week periods, a coefficient of variation for the zone event rate within each treatment of 50%, an intra-class correlation (ICC) within facilities of $\rho=0.03$ as well as a within-zone ICC of $\rho=0.20$, a total of four measurement periods per zone (two for each season) and a variance inflation factor of $VIF=(1-\rho)/4=0.2$ for the relative number of zones required in total compared with a parallel group design. Site dropouts are highly unlikely as centres have committed to the study and contingencies for facility disruption have been identified. However, additional facilities have been made available, should they be required.<br><br>Differences in infection rates between the two periods will be assessed using Poisson regression with mixed effects, with fixed effects for the treatment group, cycle, and random effects for the zone (nested within the facility), and a logarithm of the duration of the exposure for each group included as an offset term. As a sensitivity analysis, we will also assess differences in infection rates using wider time-windows for each period in order to account for the incubation period of infection. Analysis will be performed using Stata version 17. A two-sided type-1 error rate of $\alpha=0.05$ will be used to indicate statistical significance.<br><br>**Procedure Performance:** The trial will be undertaken by a highly expert collaborative research team of international standing. The team is led by a recognised expert in airway infection research, who is successfully leading MRFF-funded studies relating to both RACF infection control and COVID-19 clinical management. The team includes outstanding expertise in respiratory and rehabilitation medicine, air quality and health, provision of residential aged care, clinical trials, and health translation, as well as considerable expertise in clinical trials, bioinformatics, biostatistics and epidemiology.
**Project duration:** 3 years

9. Site Selection:

**Number of sites:** Four residential aged care facilities, with up to total n=400 beds.
**Site selection rationale:** The study settings have been selected based primarily on the ability to sub-divide resident populations into discrete areas (zones) that enable concurrent comparison of interventions in facility cohorts which are otherwise subject to the same facility practices (e.g. environmental cleaning, staffing, and social distancing). Due to the recent number of RACF COVID-19 outbreaks and guidelines distributed by SA Health (The COVID-19 Strategy for Residential Age Care Facilities), all RACFs in this study will have in place the ability to section-off zones within their facility as per Annex D of the guidelines[31].
**Inclusion and exclusion criteria:** Residential aged care facilities within South Australia

will be included if they possess the ability to sub-divide resident populations into discrete areas that enable concurrent comparison of interventions in facility cohorts which are otherwise subject to the same facility practices (e.g. environmental cleaning, staffing, and social distancing). For residents within each facility, there will be a minimum age requirement of 18 years and no limit to the maximum age, and both males and females will be included.<br><br>Residential aged care facilities within South Australia will be excluded if they do not have the ability to sub-divide resident populations into discrete areas that enable concurrent comparison of interventions in facility cohorts which are otherwise subject to the same facility practices as described above.
**Facility commitment:** All RACFs will be consulted prior to the study to outline the commitment required for involvement with the study. In summary, sites will be assessed for suitability and layout for the retrospective installation of GUV devices, a combination of units will be considered to determine the best fit. Following confirmation of layout, devices will be installed by approved certified contractors and remain in place for approximately 2 years, covering the intervention periods.<br><br>There will be several sub-groups within each RACF provider that will be involved in the study: property services/facilities, clinical surveillance team, RACF Board and/or management team. Each group will be consulted and kept up-to-date with the status and progress of the study. Regular meetings prior to the commencement of the study will be held to ensure all groups are aware of their commitment and role within the study. An RACF can opt to stop participation with the study at any time.

10. Waiver of Consent:

This study will entail an environmental intervention in the communal areas of RACFs with the aim to reduce acute respiratory infections among aged care residents. Circulating air in communal areas will be exposed to commercially available GUV devices, resulting in the inactivation of any airborne microorganisms (including viruses and bacteria). The environmental intervention will not interfere or change habits or activities of residents or staff at the RACFs. No GUV devices will be installed or used in resident rooms or other private areas, only communal areas such as corridors, dining rooms, lounges and lift lobbies will be included.

The GUV devices (LAF Technologies Pty Ltd) that will be used in the study are safe (accreditation with NATA (ISO21501-4), Eco Warranty accredited, ISO9001 Quality accredited, ISO9001:2015 certified of LightProgress production), are commercially available in Australia, and in use in multiple clinical settings. All GUV devices will be installed and maintained by a certified contractor.

The participation of residential aged care facilities in the study has been at the discretion of the provider CEOs and senior management. These staff are responsible on a daily basis for all devices, products and equipment used in communal areas. For example, the use of, HVAC systems, security cameras and environmental cleaning products.

Our proposed study conforms to the NHMRC National Statement on Ethical Conduct in Human Research guidelines for a waiver of consent as stated in Chapter 2.3.9 and 2.3.10. Below we have provided specific details:

2.3.9 Only an HREC may grant waiver of consent for research using personal information in medical research, or personal health information. Other review bodies may grant waiver of consent for other research.

Only de-identified acute respiratory illness surveillance data, and pathology results will be collected from facilities as part of this study. Therefore, the proposed application to the HREC is submitted for review to grant a waiver of consent, as identifiable data will not be collected.

2.3.10 Before deciding to waive the requirement for consent (other than in the case of research aiming to expose illegal activity), an HREC or other review body must be satisfied that:

a) involvement in the research carries no more than low risk (see paragraphs 2.1.6 and 2.1.7, page 18) to participants

The GUV devices (LAF Technologies Pty Ltd) used in the study are commercially available in Australia and NATA (ISO21501-4), Eco Warranty, ISO9001 Quality, and ISO9001:2015 certified. All GUV devices will be installed and maintained by a certified contractor. Ultraviolet rays are confined to high level room zone (UV-FLOW Germicidal), with the base of the device positioned 2.2 metres from the floor, or entirely contained within the unit (UV-FAN Germicidal; UV-FAN-XS Germicidal) when correctly installed/used, allowing air to be safely treated when persons are present. Based on the safe installation and maintenance by a certified operator there is negligible risk to aged care residents at participating facilities. All units will be tested for function and spread of UV-C light by SAHMRI researchers. This will be reported and shared with the RACF management prior to the commencement of the intervention period. A guideline document will be in place and followed at all sites to mitigate any risk of unsafe exposure to UV-C light while in operation.

The devices are not inconspicuous and as a result may concern residents, families, and care staff. To mitigate this, we will work in partnership with study sites to use previously implemented communication strategies to inform residents, families, and care staff about the study. This will include communication directly from the aged care provider via existing channels that the RACFs commonly use, information sheets, and study staff available onsite at the beginning of the study to answer questions. Each RACF will submit and prepare a Communication Action Plan for the dissemination of study information to their residents, family and staff. Only HREC-approved study brochures, information sheets and posters will be used for communications.

b) the benefits from the research justify any risks of harm associated with not seeking consent

This study has been designed as a rapid response to the COVID-19 pandemic. Aged care residents both in Australia and globally have seen some of the highest mortality rates from COVID-19. In Australia there have been a total of 2,029 cases of COVID-19 in aged care residents with approximately 33% of these individuals not surviving[30]. Once a case of COVID-19 enters an aged care facility, the exponential rise in cases has been near impossible to contain. Furthermore, this intervention will also aim to reduce several other high-risk airborne viruses including influenza and respiratory syncytial virus (RSV). Prior to the COVID-19 epidemic, in 2019, influenza was responsible for the death of 839 older Australians[30] (>65 years old). Beyond improved infection control, the only effective measure in keeping aged care residents safe has been facility closure, restricting all visitations including by immediate family members, and increased PPE, which has isn't always practical in the aged care environment where staff and residents are in close proximity and in many circumstances require physical assistance. While effective in reducing the risk of

COVID-19, this strategy further isolates older people and has its own significant impacts on health and wellbeing. We believe the potential benefits proposed by this environmental intervention, using GUV devices - already proven safe and effective – far outweighs the risk described above. In the event of a COVID-19, influenza or RSV outbreak, the intervention has the potential to significantly decrease the associated morbidity and mortality.

c) it is impracticable to obtain consent (for example, due to the quantity, age or accessibility of records)

As this study will be implementing an environmental intervention in communal areas only, we feel that it would not be practical to obtain consent for each individual resident. Firstly, for the study to run it will require 100% consent of all residents, at all times, which we believe is near impossible to achieve in this setting. In our experience, consent in residential aged care takes significant time. It often involves long conversations with residents, contacting next-of-kin, organising time to meet with next-of-kin, and in some cases finding essential documentation (power of attorney). In the context of this study the high turnover in aged care would also mean on any day there may be a new resident requiring consent. As this study is a rapid response to COVID-19 and an environmental intervention, we believe, for the reasons stated above, it is impractical to obtain individual consent. Dissemination of information regarding the study will continue and capture any new residents (and family), and will be detailed in the RACF's Communication Action Plan.

The decision for sites to participate in the study has been made by senior management for each provider, who on a daily basis make decisions about the facility and its environment and the use of equipment and devices, for the health and wellbeing of their residents.

d) there is no known or likely reason for thinking that participants would not have consented if they had been asked

In one of our recently conducted aged care studies assessing antimicrobial resistance we had a recruitment rate of 75%. The primary reasons for non-recruitment included: too unwell or unstable to be approached, no suitable third-party consent (for those with cognitive impairment) and did not wish to provide a study sample (stool sample). Nearly all eligible residents agreed to participate. Based on this experience we believe residents and their families would appreciate the significant benefit of this study and the minimal interference with their daily lives, and would be more than willing to consent.

Continued outbreaks of COVID-19 across the country and world are demonstrating the ever-present danger of the respiratory virus on vulnerable populations. We therefore believe it unlikely that participants would be unwilling to prevent a COVID-19 outbreak within their living space.

e) there is sufficient protection of their privacy

Surveillance data and pathology results of respiratory infection testing will be stored in a de-identified manner. A unique identifier will be used to record cases and pathology results. No names or dates of birth will be collected. All data will be stored securely on the SAHMRI server in password protected files/databases only accessible to study staff. Study sites will have a unique identifier and will not be identifiable in any report or publication.

f) there is an adequate plan to protect the confidentiality of data

All research data will be stored securely at SAHMRI in either a locked office or password protected in a REDCap database stored on the SAHMRI server. All study data will only be accessible to study staff. Unique identifiers will be used to deidentify study sites and respiratory illness cases.

g) in case the results have significance for the participants' welfare there is, where practicable, a plan for making information arising from the research available to them (for example, via a disease-specific website or regional news media)

All findings will be shared with the RACFs and summarised outcomes will be communicated to residents, families and staff by means of a summary report at the end of the study. In the event that there are significant findings at the end of year 1, demonstrating the benefit of GUV devices in preventing respiratory infections, results will be made immediately available in the form of an interim report to participating RACF management. A two-page summary lay interim report will also then be disseminated to residents, families and staff from participating sites.

h) the possibility of commercial exploitation of derivatives of the data or tissue will not deprive the participants of any financial benefits to which they would be entitled

No tissue samples are being collected in this study. We do not foresee any commercial exploitation of derivatives of the data. No results will identify an individual or a specific facility.

i) the waiver is not prohibited by State, federal, or international law.

A waiver of consent is not prohibited.

2.3.12 Given the importance of maintaining public confidence in the research process, it is the responsibility of each institution to make publicly accessible (for example in annual reports) summary descriptions of all its research projects for which consent has been waived under paragraphs 2.3.10 and 2.3.11.

A final study report will be disseminated to participating aged care providers, residents and families. Reports will be tailored for each audience to ensure they are accessible.

Communication: Individual RACFs will prepare a Communication Action Plan and information sheets/communication documents for each of their sites, to be shared with residents and their family; documents will be prepared with input from the research team. In addition, the SAHMRI research team will offer discussion and information sessions for staff, residents and their families to attend. All brochures, information sheets and posters will be pre-approved by HREC.

Residents with cognitive impairment, sensory impairment, non-English speaking background or from certain cultures or communities may require additional communication methods, options for translation and/or involvement with family, carers, or next of kin (National Statement 1.10, 1.13, 2.2.12, 2.2.13, 4.5). RACFs will be aware of individuals with any of these requirements and will act in accordance with the facilities standard processes.

Communication Action Plans will also include mention of methods to communicate with such groups.

Process to voice concerns:

If needed, residents can contact their facilities wellbeing or management team and/or SAHMRI researchers to discuss concerns. If the RACFs have concern they are able to contact the research team and Principal Investigator at any point.

PETRA

SAHMRI South Australian Health & Medical Research Institute

11. Data Management:

**Data collection:**<br>• **Outcome 1, 2.1, 2.4:** RACF clinical teams will follow their standard protocol for surveillance of acute respiratory illnesses and will monitor and record. These data will be used to identify events of respiratory infections (Outcome 1). Results from pathology testing (Outcome 1, 2.4) and hospital admissions (Outcome 2.1) will also be recorded, as outlined in "Methodology and Procedures". Data will be de-identified and collected and entered using REDCap, a secure database hosted at SAHMRI.<br>• **Outcome 2.2 & 2.3:** Air and fomite samples will be collected by SAHMRI researchers throughout the intervention periods as described in the "Project Design" section. All results will be stored securely on the SAHMRI server or in REDCap, also hosted on the SAHMRI server.
**Data storage:** All data will be electronically stored in REDCap, hosted by SAHMRI. All electronic data will be stored on a SAHMRI server in a password protected file. This data will be stored for 15 years following the completion of the study at which point it will be destroyed via electronic deletion. In addition, any paper-based records will be shredded 15 years after the end of the study.<br>Air and fomite study samples will be destroyed during DNA extraction. Extracted DNA will be stored in a -80°C freezer at the South Australian Health & Medical Research Institute laboratories at Flinders Medical Centre. These DNA specimens will be coded and kept in locked laboratories only accessible to the study team for analysis.
**Data archival and destruction:** All electronic data will be stored for 15 years following the completion of the study at which point it will be destroyed via electronic deletion. Paper based data, will be stored in a locked office and/or securely archived. All documentation will be shredded 15 years after the end of the study.
**Response to facility withdrawal:** RACFs will be free to withdraw at any time during the study. However, if they choose to withdraw, all samples (air and fomite) and clinical data received up to that date will be retained for analytical purposes. All GUV devices will be removed from the facility for use in a supplementary facility, if required.
**Data monitoring:** Study data will be monitored throughout the study, including internal reports, reporting to the steering committee and as required by SAHMRI governance.

12. Timeframes, Outcomes and Future Plans:

Timeframe: The study will be completed within 3 years. It will be divided broadly into four processes, the timing of which are shown below:

ACTIVITY	TIMELINE											
	Year1				Year 2				Year 3			
**Engagement, consultation, ethics**												
Stakeholder engagement	■	■										
Ethics and governance	■	■										
Intervention site-specific modification	■	■										
**Intervention and empirical data collection**												
Study intervention			■	■	■	■	■	■	■	■	■	
Environment sample collection (air/surface)			■	■	■	■	■	■	■	■	■	
Clinical/epidemiological data collection			■	■	■	■	■	■	■	■	■	
**Data processing and environmental sample analysis**												
Environmental sample processing and analysis				■	■	■	■	■	■	■	■	■
Outcome data processing and analysis				■	■	■	■	■	■	■	■	■
**Data integration and reporting**												
Reporting of interim analysis								■	■			
Data integration, final report and dissemination												■

Plans for the return of the research results to the facilities: On completion of the study, all findings will be shared with the RACFs and summarised outcomes will be communicated to residents, families and staff by means of a summary report at the end of the study. Only aggregated data will be presented ensuring no individual participant or facility can be linked to the findings. A short information sheet with key findings will also be disseminated to residents, their families, and staff, in conjunction with the release of study manuscripts. Prior to this the study team will present directly to the Board or Management team from the various providers. This presentation will provide findings at a provider and/or facility level to better inform effectiveness of GUVs and infection prevention and control policies and practice going forward. In the event that there are significant findings at the end of year 1, demonstrating the benefit of GUV devices in preventing respiratory infections, results will be made immediately available in the form of an interim report to participating RACF management.

Dissemination of project outcomes: Results from this study will be of interest to a range of sectors both nationally and internationally. Therefore, it is likely that the results are shared beyond those involved and in academic research (via peer-reviewed journals). It is planned that in addition to the aged-care sector results will also be shared with the Department of Health & Aging. At no point will information or data be shared which could identify residents. Facility level data will only be disseminated directly to the RACFs. No publicly available publications will identify facilities or providers unless permission has been granted by the facility.

Other potential uses of the data: There are no planned uses for the data collected beyond the study described.

Future use and/or follow-up research: As per NHMRC open access policy, metadata for peer-reviewed publications will be made openly accessible no later than 3 months from the date of publication. No other future use is planned.

Project closure: Final project and financial reports will be submitted to the Medical Research Future Fund within six-months of the end of the research activity date, as per the grant agreement. The final project report will also be shared with Bellberry Ltd. Human Research Ethics Committee and SAHMRI Governance at this time.

13. Risk and Monitoring

Risk Management Plan: The Risk Management Plan (**Table 1**) has been informed by SAHMRI's Risk Management Procedure. SAHMRI's Risk Rating system has been used to indicate the level of risk for each theme, considering likelihood and consequence. Management focuses on decreasing likelihood and minimising impact.

Table 1: Risk Management Plan

Risk theme	Risk	How risk is mitigated/managed
**People**	Poor stakeholder engagement (Low Risk)	The research team has a long-standing relationship with the aged care providers involved in the study and some have existing memorandum of understanding in place for previous studies.
	Pathogen exposures (Med Risk)	Processing of samples will be performed under PC2 conditions (AS/NZS 2243.3:2010 Safety in Laboratories Part 3: Microbiological Safety and Containment). Protocols have been submitted to SAHMRI IBC.
**Information**	Inadequate research data (Low Risk)	The study will take advantage of well-established surveillance data, including those required under national Communicable Diseases Network of Australia outbreak guidelines, diagnostic laboratory results and patient management information systems. Data will only be accessed retrospectively by study personnel.
	Poor data integrity (Low Risk)	Use of established data collection, entry and cleaning procedures, including an electronic data capture platform (REDCap). Storage of raw data, including air and fomite samples, securely for 15 years after study completion.
**Information**	Unplanned data disclosure (Low Risk)	Unique IDs used for data collection and sample storage. Data stored in SAHMRI's primary data centre - commercially operated, ISO27001 certified.
**Delivery**	Seasonal variation (Low Risk)	Power calculations have been based on projections of reduced respiratory viral infection rates. Additional facilities have also been included to account for this event. Where case numbers and seasonal variations deviate from the projected rates within the study

		population, the experimental schedule will be extended to capture these changes.
	Research integrity (Low Risk)	Use of well-established and validated methods for all proposed work.
	Facility closure/changes in IPC/facility access (Med Risk)	Facility closures due to infectious outbreaks including SARS-CoV-2 may occur during the trial. In the instance this occurs before commencement of the study it may result in delayed installation of GUVs. Once installed GUVs will be managed by the facility engineer so the event of an outbreak and a facility lockdown should not impede the progress of the trial. Monitoring data will capture changes in IPC, environmental cleaning practices and any facility restrictions imposed by the individual facility or state government. These data will be used to contextualise the results.
	Engineering failure (Med Risk)	Multiple facilities have been included to account for unplanned technical failures, for example a power outage, which would cause the GUV devices to become redundant.
	Innovation (Low Risk)	While a highly innovative proposal, no methodological development is required to achieve the study outcomes. The GUV devices are commercially available and have been effectively used in other healthcare settings. The GUV devices are supplied by LAF Technologies, which is ISO90001 quality accredited, Eco warranty accredited and NATA accredited to ISO 17025.
	Performance measures not met (Low Risk)	Progress towards the study milestones tracked by the PI and the study manager. Progress against the milestones included in annual reports and reported to the study steering committee.
	Financial mismanagement (Med Risk)	The study will follow institutional financial procedures, including financial oversight from SAHMRI's Chief Financial Officer.
	Poor practice / analysis (Low Risk)	Analysis will be conducted according to the predefined statistical analysis plan. Raw data, including samples, will be retained for 15 years after study completion.
**Regulatory & Governance**	Legislation noncompliance (Low Risk)	PI laboratories adhere to national legislative frameworks and have Institutional Biosafety Committee approval to conduct the work required.
**Regulatory & Governance**	Ethics noncompliance (Low Risk)	The study will comply with all aspects of the NHMRC *Australian Code for the Responsible Conduct of Research* and *NHMRC – Ethical conduct in research Aboriginal and Torres Strait Island Peoples and*

		communities. Ethics approvals will be obtained from Bellberry Ltd. Human Research Ethics Committee.
	Policy noncompliance (Low Risk)	Research will be conducted according to SAHMRI's Responsible Conduct of Research policy 2019 and consistent with local policies and practice, as advised.
	Reporting (Low Risk)	The study will produce annual reports for the study steering committee, MRFF and ethics committees. Dissemination of final study reports, including community reports, will be done via established networks.

COVID-19 Risk Management Plan: All members of the SAHMRI research team will follow any current guidelines[31,32,33] required to enter a RACF. Updates and guidelines by both SA Government and individual RACFs will be adhered to. As of July 2021, all study staff must provide evidence of the current season's influenza vaccine and follow all declarations and sign-in/out procedures upon entry to a site.

The following table (**Table 2**) outlines the possible scenarios, the potential impacts on the study, and mitigation measures to be taken in response to COVID-19.

Persons responsible: Principal Investigator (PI), Study Manager (SM), Associate Investigator (AI).

Table 2: COVID-19 Risk Management Plan

Scenario	Risk	Associated harm	Mitigation measures	Responsible person
**Lockdown at RACF**	Installation of GUV units delayed	Start of study delayed	• GUV devices installed as soon as available.	SM<br>AI
	Study staff unable to come onsite	Staff unable to perform ongoing study task, including environmental sample collection.	• GUV units will continue to follow study schedule, SAHMRI staff can communicate with RACF maintenance staff to turn on or off the units.<br>• Maintenance team leader for each RACF is trained on how to turn on and off the GUV units.<br>• Collection of clinical data such as the number of cases and admittance to hospitalisation (due	SM<br>AI

			to acute respiratory infection) can continue to be recorded remotely (e.g. telephone).<br>• Collection of environmental samples (air and surface) will cease during any lockdown situations.	
	RACF partners overwhelmed with COVID-19 related tasks	Partners unable to perform study related tasks	• Collection of clinical data such as the number of cases and admittance to hospitalisation (due to acute respiratory infection) postponed until a suitable time to share the data.	SM
**Visitor restrictions at RACF**	Study staff unable to come onsite	Staff unable to perform ongoing study task, including environmental sample collection.	• GUV units will continue to follow study schedule, SAHMRI staff can communicate with RACF maintenance staff to turn on or off the units.<br>• Maintenance team leader for each RACF is trained on how to turn on and off the GUV units.<br>• Collection of clinical data such as the number of cases and admittance to hospitalisation (due to acute respiratory infection) can continue to be recorded remotely (e.g. telephone).	SM<br>AI

			• Collection of environmental samples (air and surface) will cease during any lockdown situations.	
	RACF partners overwhelmed with COVID-19 related tasks	Partners unable to perform study related tasks	• Collection of clinical data such as the number of cases and admittance to hospitalisation (due to acute respiratory infection) postponed until a suitable time to share the data.<br>• Collection of environmental samples (air and surface) collected when restrictions ease.	AI
**Study staff member exposed to COVID-19 case or exposure site**	RACF partner site a secondary contact	RACF partners alarmed	• Study staff aware of current exposure sites.<br>• Adherence with SA Health's instructions.<br>• Prompt and transparent communication with study partners.<br>• All staff to receive a COVID-19 vaccination before coming onsite.<br>• Staff do not attend with cold or flu like symptoms.<br>• Staff to use check-in systems at all activities.	PI
	Key staff members	Staff unable to perform ongoing study	• Multiple staff trained in the collection of	SM<br>AI

	unable to come onsite	task, including environmental sample collection	environmental samples.<br>• Collection of clinical data such as the number of cases and admittance to hospitalisation (due to acute respiratory infection) can continue to be recorded remotely (e.g. telephone).	
**Lockdown at SAHMRI due to COVID-19 case**	RACF partner site a secondary contact	RACF partners alarmed	• Follow SAHMRI COVID-19 guidelines.<br>• Prompt communication with the study partners<br>• All staff to receive a COVID-19 vaccination before coming onsite.<br>• Staff do not attend with cold or flu like symptoms.<br>• Staff to check-in using QR codes for all activities.	PI
	Key staff members unable to come onsite for an extended period	Staff unable to perform ongoing study task, including environmental sample collection.	• GUV units will continue to follow study schedule, SAHMRI staff can communicate with RACF maintenance staff to turn on or off the units.<br>• Maintenance team leader for each RACF is trained on how to turn on and off the GUV units.<br>• Collection of clinical data such as the number of cases and	SM<br>AI

			admittance to hospitalisation (due to acute respiratory infection) can continue to be recorded remotely (e.g. telephone).<br>• Key RACF staff member trained via Zoom on the collection of environmental samples	
**State or Adelaide wide short "circuit-breaker" lockdown**	Study staff unable to go onsite for a short period	Staff unable to perform ongoing study task, including environmental sample collection.	• GUV units will continue to follow study schedule, SAHMRI staff can communicate with RACF maintenance staff to turn on or off the units.<br>• Maintenance team leader for each RACF is trained on how to turn on and off the GUV units.<br>• Collection of clinical data such as the number of cases and admittance to hospitalisation (due to acute respiratory infection) can continue to be recorded remotely (e.g. telephone).<br>• Collection of environmental samples (air and surface) will take place as soon as feasible.	SM<br>AI
**State or Adelaide outbreak and months**	Study staff unable to go onsite for an	Staff unable to perform ongoing study task, including	• GUV units will continue to follow study schedule, SAHMRI staff can	SM<br>AI

**long lockdown**	extended period	environmental sample collection.	communicate with RACF maintenance staff to turn on or off the units.<br>• Maintenance team leader for each RACF is trained on how to turn on and off the GUV units.<br>• Collection of clinical data such as the number of cases and admittance to hospitalisation (due to acute respiratory infection) can continue to be recorded remotely (e.g. telephone).<br>• Key RACF staff member trained via Zoom on the collection of environmental samples	

Risk Monitoring: PI and the Study Manager will monitor study progress based on the defined milestones and performance indicators. This assessment will be undertaken on a weekly basis in order to capture any new risks arising from changing circumstances. High and Extreme Risks will be immediately reported to the study steering committee in accordance with the SAHMRI Risk Management Procedure. The Risk Management Plan will be reported quarterly to the study steering committee and annually to SAHMRI governance and the MRFF. Changes to identified risks, including new or emerging risks, will be highlighted.

14. Study Governance:

Study oversight and governance: The study will comply with the NHMRC *Australian Code for the Responsible Conduct of Research* and an agreement between each RACF and SAHMRI will be established. A Protocol Steering Committee will be established to monitor trial progress and its ongoing ability to meet the stated objectives. Risk and financial management will comply with SAHMRI's institutional policies. Consumer involvement will continue to be gathered through existing consumer reference groups at SAHMRI.

15. References:

1. Australian New Zealand Clinical Trials Registry, 2020. (https://www.anzctr.org.au/Trial).
2. ClinicalTrials.gov, U.S. National Library of Medicine. 2020. (https://clinicaltrials.gov/)
3. Crotty F et al. Intern Med J. 2020. doi:10.1111/imj.14966
4. UK Government. 2020 (https://www.gov.uk/government/publications/coronavirus-covid-19-admission-and-care-of-people-in-care-homes)
5. World Health Organization, 2020 (www.who.int/news-room/commentaries/ detail/transmission-of-sars-cov-2-implications-for-infection-prevention-precautions).
6. Shen Y et al. JAMA Intern Med. 2020. doi:10.1001/jamainternmed.2020.5225
7. Liu Y et al. Nature 2020:582 (7813), 557-560.
8. Lindsley WG et al. PLoS One 2010:5 (11), e15100.
9. Agranovski IE et al. Atmos Environ 2004:38 (23), 3879-3884.
10. Morawska L et al. Environ Int 2020:142:105832.
11. McDevitt JJ et al. Appl Environ Microbiol 2012:78 (6), 1666-9.
12. Darnell ME et al. J Virol Methods 2004:121 (1), 85-91.
13. Buonanno M et al. Sci Rep 2020:10 (1), 10285.
14. McDonald CJ. mSystems 2020: 5 (3), e00390-20
15. Mphaphlele M et al. Am J Respir Crit Care Med 2015:192 (4), 477-84.
16. Menzies, D. et al. Lancet 2003:362 (3), 1785-91.
17. Nardell EA et al. JAMA 2020:324 (2), 141-142.
18. Alsved M et al. Clinical Infectious Diseases 2020:70 (10), 2023-2028.
19. Bailey ES et al. Tropical diseases, travel medicine and vaccines 2018:4;11.
20. Australian Institute of Health and Welfare 2016. Australian Burden of Disease Study: Impact and causes of illness and death in Australia 2011. Canberra: AIHW
21. Australian Government Department of Health, 2020. https://www.health.gov.au/resources/covid-19-cases-in-aged-care-services-residential-care
22. Commission Internationale de l'Éclairage. Ultraviolet Air Disinfection, CIE Vienna, 2003.
23. Australian Bureau of Statistics. Regional Population by Age and Sex, Australia, 2019.
24. Australian Government Department of Health. 2019. https://www.health.gov.au/ resources/ publications/2018-19-report-on-the-operation-of-the-aged-care-act-1997
25. Australian Government Department of Health, 2022. https://www.health.gov.au/sites/default/files/documents/2022/09/national-guidelines-for-the-prevention-control-and-public-health-management-of-outbreaks-of-acute-respiratory-infection-including-covid-19-and-influenza-in-residential-care-facilities.pdf
26. Brittain-Long R et al. Journal of Clinical Virology 2010:47 (3), 263-267.
27. Mizumoto K et al. Euro Surveill 2020:25 (10), 2000180.

28. Yang W et al. J R Soc Interface 2011:8 (61), 1176-84.
29. Fabian P et al. J Aerosol Med Pulm Drug Deliv 2011:24 (3), 137-47.
30. Julian TR et al. Appl Environ Microbiol 2011:77 (19), 6918-25.
31. Australian Government Department of Health, 2021. https://www.health.gov.au/resources/publications/covid-19-outbreaks-in-australian-residential-aged-care-facilities-7-may-2021.
32. SA Health, Aged Care - COVID-19: https://www.sahealth.sa.gov.au/wps/wcm/connect/public+content/sa+health+internet/conditions/infectious+diseases/covid-19/response+and+restrictions/aged+care+facility+visitation+in+south+australia+-+covid-19
33. SA Health, Contact Tracing – COVID-19: https://www.sahealth.sa.gov.au/wps/wcm/connect/public+content/sa+health+internet/conditions/infectious+diseases/covid-19/testing+and+tracing/contact+tracing/contact+tracing
