# Supplementary material for: Germicidal UV Light and Incidence of Acute Respiratory Infection in Long-Term Care for Older Adults: A Randomized Clinical Trial
Source: JAMA Intern Med. 2025 Jul 28;185(9):1128–35. doi: 10.1001/jamainternmed.2025.3388 (PMC12305439; doi:10.1001/jamainternmed.2025.3388)
Supplement: Supplement 2. — Statistical analysis plan [file jamainternmed-e253388-s002.pdf]

Statistical Analysis Plan

The PETRA Study:

Prevention of SARS-CoV-2 (COVID-19) Transmission in Residential Aged Care using ultraviolet light (PETRA): a parallel crossover randomised controlled trial

Version: 4.

Version Date: 22nd January, 2025

Based on

Protocol Version: 5.

Protocol Date: 26th September, 2022

Title: Prevention of SARS-CoV-2 (COVID-19) Transmission in Residential Aged Care using ultraviolet light (PETRA): a parallel crossover randomised controlled trial.

Trial Registration: ACTRN12621000567820 (https://anzctr.org.au/).

Funding: The trial was supported by a Medical Research Future Fund grant from the Australian Government (GNT2016047).

Signatures: By signing this document, I am confirming that I have read, understood and approve this statistical analysis plan (SAP).

Dr Andrew Shoubridge – Investigator

Signature: Date: 22/01/2025

Professor Geraint Rogers – Principal Investigator

Signature: Date: 23/01/2025

Professor Richard Woodman – Trial Statistician

Signature: Date: 23/01/2025

Version History:

SAP Version	Protocol Version	Section Amended	Reason for Amendment	Date of Amendment
Version 1	Version 4	-	New document	-
Version 2	Version 4	6.1	Expanded testing methods in response to development of RATs	23/12/2021
Version 3	Version 5	4, 8.1, 8.3, 8.8	Increase in cycle number	26/09/2022
Version 4	Version 5	13.1	Updated Stata code to reflect increase in cycle number	22/01/2025

Table of Contents

1. Introduction
2. Study Aims and Objectives
3. General Study Design
4. Intervention
5. Randomisation, Allocation and Blinding
6. Outcome Measures
    - 6.1. Primary Outcome
    - 6.2. Secondary Outcomes
7. Sample Size and Power Calculation
8. Statistical Methods
    - 8.1. General Methods
    - 8.2. Interim Analysis
    - 8.3. Primary Outcome
    - 8.4. Secondary Outcomes
    - 8.5. Subgroup Analyses
    - 8.6. Sensitivity Analysis
    - 8.7. Missing Data
        - 8.7.1. Missing Baseline Data
        - 8.7.2. Missing Outcome Data
    - 8.8. Statistical Software
9. Effect of COVID-19
10. Proposed Tables
11. Proposed Figures
12. References
13. Appendix
    - 13.1 Appendix 1: Stata code

PETRA

This is the statistical analysis plan (SAP) detailing the statistical analyses planned for PETRA. The SAP is based on the PETRA protocol and any deviations from the plan will be described.

1 Introduction

Residential aged care facilities (RACF) have experienced catastrophic outbreaks of COVID-19. Rapid transmission of SARS-CoV-2 within facilities, combined with the increased likelihood of severe illness or death due to resident age, comorbidities and frailty, resulted in the highest mortality rate of any population (29.5%). Multiple separate outbreaks occurred in Australian residential aged care facilities, despite increasing efforts to prevent SARS-CoV-2 transmission, including by limiting facility access, preventing staff from working at multiple sites, using personal protective equipment, increasing hand hygiene and environmental cleaning, and implementing social distancing. It is critically important that more effective strategies to protect those in aged care from COVID-19 are identified rapidly.

Given the scale of the threat posed by COVID-19 to aged care residents, it is imperative that the potential for airborne transmission in these settings is mitigated. The Prevention of COVID-19 Transmission in Residential Aged Care using ultraviolet light (PETRA) study will determine the impact of rapidly deployable, cost-effective measures to reduce airborne viral transmission in RACF settings. These interventions will be assessed in a world-first multicentre randomised cluster-controlled trial of air-sterilisation strategies.

2 Study Aims and Objectives

Aim: The aim of this trial is to evaluate the efficacy of retrofitted commercially available GUV light devices in reducing rates of airborne respiratory viral transmission in long-term aged care settings.

Primary Objective: The primary objective is to deploy germicidal ultraviolet (GUV) light in communal spaces within RACFs, and determine if they can reduce the incidence of acute respiratory infections (ARIs), including SARS-CoV-2.

Secondary Objective: The secondary objectives are to measure the impact of GUV light on rates of related hospitalisations, respiratory virus detection in air samples, respiratory virus detection in fomite samples, and genomic characteristics of viral samples.

3 General Study Design

Type of Design: PETRA is designed to assess the effect of GUV light devices in common spaces of RACFs on the incidence of acute respiratory infections. This study is a multicentre, pragmatic, cluster randomised controlled trial utilising a two-arm double crossover, non-blinded design.

Sample Size: Based on a randomised 4-period parallel cross-over control design in which each RACF contains zones assigned to both the intervention and the control condition twice (once for each condition in each of two consecutive respiratory infection seasons), we calculate that a sample size of n=8 zones (across four facilities, from three aged care providers), with an average size of n=40 residents per zone, will provide 89% power to detect a 50% reduction in rate of symptomatic infections i.e. five per 1000 person-days in the intervention group versus 10 per 1000 person-days in the control group. This calculation assumes an average of 35 days of follow-up for each resident for each of the six-week periods, a coefficient of variation for the zone event rate within each treatment of 50%, an intra-class correlation (ICC) within facilities of $\rho=0.03$ as well as a within-zone ICC of $\rho=0.20$, a total of four measurement periods per zone (two for each season) and a variance inflation factor of $VIF=(1-\rho)/4=0.2$ for the relative number of zones required in total compared with a parallel group design.

Study Hypothesis: We hypothesise that facility-tailored GUV strategies, as an adjunct to existing infection control measures, can substantially reduce rates of acute respiratory infection in RACF residents.

4 Intervention

Intervention Type: Commercially available GUV light appliances (Laftech; LAF technologies, Melbourne, Australia) will be implemented to sterilise air. Specifically, a combination of UV-FLOW-C wall- and ceiling-mounted systems, UV-FAN M2/95HP and

UV-FAN-XS wall-mounted air purifiers (LAF Technologies, Melbourne, Australia) will be utilised.

Intervention Duration: Each facility contains zones assigned to both the intervention (GUV activity involving wall-mounted fan-driven and passive GUV devices within common areas) and the control condition (no GUV activity) for each cycle (once for each condition in each of the study cycles). The intervention will include a treatment and a control/comparator arm:

- Arm 1 (intervention): The GUV intervention will be applied continuously for a six-week period, followed by a two-week washout period (to account for respiratory virus incubation periods), and then a crossover to the control period for six-weeks, followed by a final two-week washout.
- Arm 2 (control): The control period will be applied for six-weeks, followed by a two-week washout period, and then a crossover to the GUV intervention will apply continuously for six-weeks, followed by a final two-week washout.

The study schedule will be implemented for a total of seven consecutive cycles, commencing on 31 August, 2021, and encompassing two full winter respiratory virus seasons.

5 Randomisation, Allocation and Blinding

Concealed random allocation from a computer-generated random numbers table will be used to start the control/intervention order within the crossover study design. Each zone represents the unit of randomisation and the level of intervention delivery. Zones are paired within facilities, with one zone in each pair randomised to the intervention or control condition for the first cycle. Each zone is randomly allocated to receive GUV light air treatment, or no air treatment, for the six-week duration of the first period. Once allocated, the six-week periods are separated by a two-week 'washout' period, where all devices are off, before cross over to the reciprocal condition.

Zones are arranged to simplify the operational logistics of delivering an intervention that accommodates different building characteristics and layouts. This arrangement also obviates the need for individual consent from all residents within the LTCF, and also enables everyone in the cluster to be treated in the same way [4].

Residents and care providers were not blinded to the intervention. The trial statistician will be blinded to randomisation sequence throughout the analysis.

6 Outcome Measures

6.1 Primary Outcome

The primary outcome is the incidence rate ratio of combined acute respiratory infections, as defined according to national (Communicable Disease Network Australia) [1] or local authority (Communicable Diseases Control Branch of South Australia) [2] frameworks (which are based on European guidelines [3]), for the intervention arm versus the control arm. They define acute respiratory infection clinical symptoms as the recent onset of new or worsening: cough, breathing difficulty, sore throat, nasal congestion, or runny nose. During the study periods, respiratory infections will be counted where residents meet the clinical symptomatic definition of an acute respiratory infection and/or test positive for an acute respiratory virus through routine screening or diagnostic testing. Testing will be in the form of rapid antigen tests (RATs) for SARS-CoV-2, or respiratory viral multiplex PCR panels for the following pathogens: adenovirus, *B. pertussis*, influenza A, influenza B, HMPV, *M. pneumoniae*, parainfluenza 1, parainfluenza 2, parainfluenza 3, RSV, or SARS-CoV-2.

6.2 Secondary Outcomes

Secondary Outcome 1: Rates of hospitalisation for complications associated with respiratory infection. Hospital admissions, or presentations at hospital emergency departments for complications associated with acute respiratory infection, will be recorded through facility notes where possible, including viral diagnostics.

Secondary Outcome 2: Respiratory virus detection and quantification in facility air samples. Levels of viral particles in air will be determined using quantitative, non-multiplexed versions of PCR assays.

Secondary Outcome 3: Rate of respiratory viral detection and quantification in facility fomite samples. Levels of viral particles on fomite samples will be determined using quantitative, non-multiplexed versions of PCR assays.

Secondary Outcome 4: Genomic characteristics of respiratory illnesses. Data will be captured on specimens sent to SA Pathology for respiratory virus testing during the study

period. Viral nucleic acid will be extracted and stored by SA Pathology for all positive specimens. Viral sequencing will be undertaken to determine epidemiological links.

7 Sample Size and Power Calculation

The trial sample size is for a randomised 4-period double crossover control design, in which each RACF contains zones assigned to both the intervention and the control condition twice (once for each condition in each of two consecutive respiratory infection seasons). As there are no accurate estimates for incident rate of COVID-19, historical influenza rates have been utilised as a reference. On this basis, a sample size of n=8 zones (across four facilities, from three aged care providers), with an average size of n=40 residents per zone, is estimated to provide 89% power to detect a 50% reduction in rate of symptomatic infections i.e. five per 1000 person-days in the intervention group versus 10 per 1000 person-days in the control group. This calculation assumes an average of 35 days of follow-up for each resident for each of the six-week periods, a coefficient of variation for the zone event rate within each treatment of 50%, an intra-class correlation (ICC) within facilities of ρ=0.03 as well as a within-zone ICC of ρ=0.20, a total of four measurement periods per zone (two for each season) and a variance inflation factor (VIF) =(1-ρ)/4=0.2 for the relative number of zones required in total compared with a parallel group design.

8 Statistical Methods

8.1 General Methods

RACFs within metropolitan and regional South Australia will be recruited if they are able to sub-divide communal living areas into discrete areas (zones) that enable a concurrent comparison of interventions in cohorts that are otherwise subject to the same facility infection control practices. Final analysis will take place following final data collection, with all results presented following the standard CONSORT recommendations. Descriptive analysis will be performed using means and standard deviations for normally distributed variables, medians and interquartile ranges (IQR) for non-normally distributed variables and frequencies (percentage) for categorical variables. Overall incidence rates for infections will be calculated as the mean (95% confidence interval [CI]) number of cases per zone per cycle.

Modification of the original trial design [5] is necessitated by events external to the study. Most notably, the study will be extended from two to seven cycles (continuous for the study duration).

8.2 Interim Analysis

In the event that there are significant findings at the end of year 1, demonstrating the benefit of GUV devices in preventing ARIs, results will be made immediately available in the form of an interim report to participating RACF management. The statistician will remain blinded to all groups during this interim analysis.

8.3 Primary Outcome

Difference in infection rates between the two periods will be assessed using mixed effects logistic regression with fixed effects for the treatment group, treatment order, intervention period and a period-to-treatment interaction term in order to assess for a possible treatment-to-period interaction effect. The outcome will be the weekly number of infections occurring in each separate treatment arm within each facility/zone and cycle. The zone and facility will be included as random intercepts with zones nested within facilities. As a sensitivity analysis, we will also assess differences in infection rates using wider time-windows for each period in order to account for the incubation period of infection. A two-sided type-1 error rate of alpha=0.05 will be used to indicate statistical significance.

Due to the extended nature of the study that will last a total of 110 weeks between the beginning of the first cycle and the end of the final cycle, we will also assess the rate of increase of the cumulative number of infections using time-series regression, and the difference in the increase by experimental condition. Autoregressive (AR) modelling will be used to remove the autocorrelation in residuals from an ordinary least-squares (OLS) regression model by adding appropriate autocorrelation parameters. Stationarity of the time-series will then be assessed for OLS regression and AR models using the Durbin-Watson (DW) test for autocorrelation and generation of autocorrelation function (ACF) plots, partial autocorrelation (PACF) plots, white noise series plots, standardised residual plots and residual normality plots. For the DW test, the null hypothesis of a unit root and stationarity of the time series (no autocorrelation) is rejected if $p<0.05$. The autoregressive model is formulated as:

$$y_t = x_t'\beta + \nu_t$$

$$\nu_t = -\varphi_1 \nu_{t-1} - \varphi_2 \nu_{t-2} - \cdots - \varphi_m \nu_{t-m} + \varepsilon_t$$

ε_t ~ IN (0, σ^2), which indicates that each ε_t is independently and normally distributed with mean 0 and variance σ^2.

8.4 Secondary Outcomes

Analysis for hospitalisations and respiratory viruses will be broadly the same as for the primary outcome. A mixed effects Poisson regression model will be used with the weekly number of hospitalisations/viruses in each facility/zone/cycle used as the dependent outcome. Zone will be included as a random intercept, and fixed effects will include either treatment arm alone, or treatment arm, cycle and a group × cycle interaction to assess the presence of heterogeneity of the treatment arm effect by cycle. Depending on the variance of the data and the prevalence of zeroes, the use of a Poisson regression model will be changed to either a negative binomial or zero-inflated Poisson regression to achieve better model fit.

8.5 Subgroup Analyses

We will perform a subgroup analysis amongst those facilities and zones that are not MSUs. An MSU provides specialist care environments to those with behavioural and psychological symptoms of dementia.

8.6 Sensitivity Analysis

For sensitivity analyses we will examine the number of infections recorded using a three-day lag period that allows for the possibility of infections having occurred up to three days before symptoms are experienced and reported in either the control, intervention, or washout period. This will result in exclusion of cases reported within the first three days of a control/intervention/washout period and inclusion of cases reported up to three-days following a control/intervention/washout period.

8.7 Missing Data

8.7.1 Missing Baseline Data

We do not consider that there is a potential for missing baseline data since no individual patient data will be used in the analysis. The zones and cycles to be used in the analysis are

known in advance, and therefore the only potential for missing data will be for the outcome which is the weekly number of infections occurring after baseline (see next section).

8.7.2 Missing Outcome Data

Since infection counts will be recorded for each week of the study, there should be no known missing data to be accounted for beyond the infections that may be missed. These can be assumed as occurring randomly between the two arms and across the study period since there is no known mechanism that would support bias in data reporting between arms. We will not therefore rely on the use of our mixed effects models to provided unbiased estimates of the treatment effects should any missing (unobserved) infections occur that would result in measurement error.

8.8 Statistical Software

Mixed effects Poisson regression will be performed in Stata (version 17.0). Time-series analysis will be performed in SAS. Statistical significance for all hypothesis testing will be set using a 2-sided type 1 error rate of $\alpha=0.05$.

9 Effect of COVID-19

For a complete risk management plan of COVID-19, see Table 2 of the PETRA Protocol. In brief, facility closures due to infectious outbreaks including SARS-CoV-2 may occur during the trial. In the instance this occurs before commencement of the study it may result in delayed installation of GUVs. Once installed GUVs will be managed by the facility engineer so the event of an outbreak and a facility lockdown should not impede the progress of the trial. Monitoring data will capture changes in IPC, environmental cleaning practices and any facility restrictions imposed by the individual facility or state government. These data will be used to contextualise the results. For any interruptions during the study, use of the intervention will proceed to meet the primary outcome, however, data for the secondary outcomes may not be able to be collected.

10 Proposed Tables

Table 1 – Facility characteristics

Facility characteristic	Facility 1	Facility 2	Facility 3	Facility 4
Organisation Type				
Service Provider				
Location				
Beds available				
Beds occupied, mean (%)				

Table 2 – Resident and respiratory infection characteristics

Resident characteristic	Facility 1	Facility 2	Facility 3	Facility 4
Acute respiratory infection events, No. (%)				
Age, median (IQR)				
Sex, No. (%)				
Female				
Male				
Located in memory support area, No. (%)				
**Symptom onset, No. (%) (not mutually exclusive)**				
Cough (new or worsening)				
Sore throat				
Shortness of breath				
Fever or feverishness				
Malaise				
Headache				
Myalgia				
Runny or congested nose				
Loss of taste or smell				
Fatigue				
Nausea, vomiting, or diarrhoea				
Loss of appetite				

Table 3 – Respiratory infection incidence rates

Condition	Recorded events (n)	Infections per zone per cycle Mean (95% CI)	Incidence rate ratio (95% CI)	Estimated mean difference in infections per zone per cycle (95% CI) (Treatment vs Control)	P-value
Control					
Intervention					
Control (excl. MSU)					
Intervention (excl. MSU)					

Table 4 – Cumulative incidence of respiratory infections

Condition	Increase in infections per week β (95% CI)	Difference in infections (95% CI) per week (P-value: Treatment versus Control)
**All beds**		
Control		
Intervention		
AR1		
AR2		
**Excluding MSU beds**		
Control		
Intervention		
AR1		
AR2		

AR1, AR2= First and second order autoregressive parameters in autoregressive time-series analysis.

Table 5 - Respiratory infection incidence rates after sensitivity adjustments

Adjustment	Condition	Recorded events (n)	Infections per zone per cycle Mean (95% CI)	Incidence rate ratio (95% CI)	Estimated mean difference in infections per zone per cycle (95% CI) (Treatment vs Control)	P-value
Three-day incubation window	Control					
	Intervention					
MSU exclusion with three-day incubation window	Control					
	Intervention					

Table 6 – Cumulative incidence of respiratory infections after sensitivity adjustments

Adjustment	Condition	Increase in infections per week β (SE)	Difference in slopes (SE) (Δ cases per week) (P-value: Treatment versus Control)
Three-day incubation window	Control		
	Intervention		
	AR1		
	AR2		
MSU exclusion with three-day incubation window	Control		
	Intervention		
	AR1		
	AR2		

AR1, AR2= First and second order autoregressive parameters in autoregressive time-series analysis.

Table 7 – Infection control practices of participating RACFs

Baseline infection control practice	Facility 1	Facility 2	Facility 3	Facility 4
Staff with season's influenza vaccination, %				
Staff with one dose COVID-19 vaccination, %				
Staff with two doses COVID-19 vaccination, %				
Surgical masks worn by staff				
Face shields worn by staff				
Restrictions on resident movements				
Visitation restrictions				
Visitors screened (temperature and influenza vaccination)				
Frequency of touch-points disinfected				
Residents with season's influenza vaccination, %				
Residents with one dose COVID-19 vaccination, %				
Residents with two doses COVID-19 vaccination, %				

11 Proposed Figures

Figure 1 – CONSORT Flow Diagram

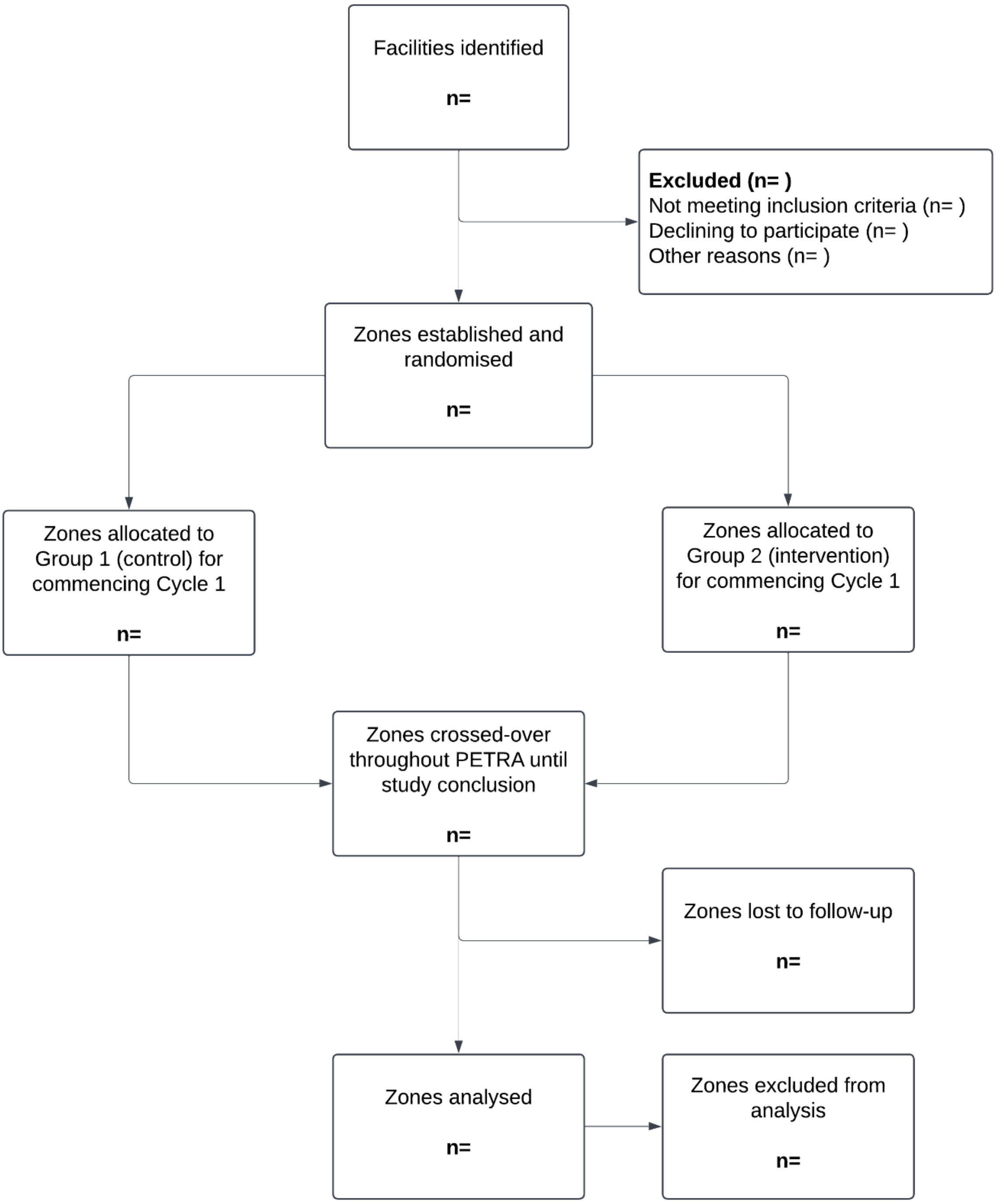

Figure 2 – Study cycle design

Figure 3 – Layout of GUV devices across a RACF zone

Figure 4 – Incidence rates of respiratory infections by group

Figure 5 – Cumulative incidence of respiratory infections by group

Figure 6 – Cumulative incidence of respiratory infections by group, excluding MSUs

Figure 7 – Cumulative incidence of respiratory infection by group, after sensitivity adjustments

Figure 8 – Changes in state and national infection control practices during study

Figure 9 – Changes in RACF infection control practices during study

12 References

1. National Guideline for the Prevention, Control and Public Health Management of Outbreaks of Acute Respiratory Infection in Residential Aged Care Homes. Australian Government. https://www.health.gov.au/resources/publications/national-guidelines-for-the-prevention-control-and-public-health-management-of-outbreaks-of-acute-respiratory-infection-in-residential-care-facilities
2. Infectious disease control. Government of South Australia. https://www.sahealth.sa.gov.au/wps/wcm/connect/public+content/sa+health+internet/clinical+resources/clinical+programs+and+practice+guidelines/infectious+disease+control
3. WHO. Prevention and control of outbreaks of seasonal influenza in long-term care facilities: a review of the evidence and best-practice guidance. World Health Organization. https://iris.who.int/bitstream/handle/10665/375205/WHO-EURO-2017-8670-48442-71937-eng.pdf?sequence=1&isAllowed=y
4. Hemming K, Taljaard M. Key considerations for designing, conducting and analysing a cluster randomized trial. Int J Epidemiol. 2023;52(5):1648-58.
5. Brass A, Shoubridge AP, Crotty M, Morawska L, Bell SC, Qiao M, et al. Prevention of SARS-CoV-2 (COVID-19) transmission in residential aged care using ultraviolet light (PETRA): a two-arm crossover randomised controlled trial protocol. BMC Infect Dis. 2021;21(1):967.

13 Appendices

13.1 Stata Code

Outcome	Stata Code
Primary Outcome:	Acute respiratory infection without lag
	**#Overall IRR between treatment arms**<br>`mepoisson fail_nolag i.Group, irr nolog exposure(bed_days) || zone:`<br>`margins Group`<br><br>**#Test for interaction effect between treatment arm and cycles**<br>`mepoisson fail_nolag Group##cycle, irr nolog exposure(bed_days) || zone:`<br>`testparm Group#cycle`<br>`margins Group, contrast(cieffects) post`<br><br>**#Estimated IRR (95% CI) by cycle**<br>`mepoisson fail_nolag Group##cycle, irr nolog exposure(bed_days) || zone:`<br>`margins Group, at(cycle = (1 2 3 4 5 6 7))  contrast(cieffects) post`
