# Supplementary material for: Germicidal UV Light and Incidence of Acute Respiratory Infection in Long-Term Care for Older Adults: A Randomized Clinical Trial
Source: JAMA Intern Med. 2025 Jul 28;185(9):1128–35. doi: 10.1001/jamainternmed.2025.3388 (PMC12305439; doi:10.1001/jamainternmed.2025.3388)
Supplement: Supplement 3. — eMethods. Trial design, participants, interventions, outcomes, randomisation, statistical methods, and statistical software eFigure 1. Example deployment of GUV appliances across a LTCF zone eFigure 2. PETRA trial stages eFigure 3. Respiratory virus circulation in South Australia (SA) corresponding with changes to national and state-wide changes in infection control practices eTable 1. Baseline infection control practices of enrolled long-term aged care facilities at commencement of PETRA eTable 2. Characteristics of residents with reported acute respiratory infection events by facility during the control or intervention periods eFigure 4. Incidence of respiratory infections in PETRA in response to changes in facility infection control practices eTable 3. Cumulative incidence of respiratory infections in control versus intervention conditions eTable 4. Number of infections and incidence rate ratio (IRR) for acute respiratory infections after sensitivity adjustments eTable 5. Number of infections and incidence rate ratio (IRR) for acute respiratory infections by zone after sensitivity adjustments eFigure 5. Cumulative incidence of respiratory infections in control versus intervention after sensitivity adjustments and exclusions eTable 6. Estimated increase in infections per week for residents in intervention versus control groups after sensitivity adjustments and exclusions eTable 7. Incidence of public hospitalisation for complications associated with respiratory infection in intervention versus control groups [file jamainternmed-e253388-s003.pdf]

Supplemental Online Content

Shoubridge AP, Brass A, Crotty M, et al. Germicidal UV light and incidence of acute respiratory infection in long-term care for older adults: a randomized clinical trial. *JAMA Intern Med*. Published online July 28, 2025. doi:10.1001/jamainternmed.2025.3388

eMethods. Trial design, participants, interventions, outcomes, randomisation, statistical methods, and statistical software

eFigure 1. Example deployment of GUV appliances across a LTCF zone

eFigure 2. PETRA trial stages

eFigure 3. Respiratory virus circulation in South Australia (SA) corresponding with changes to national and state-wide changes in infection control practices

eTable 1. Baseline infection control practices of enrolled long-term aged care facilities at commencement of PETRA

eTable 2. Characteristics of residents with reported acute respiratory infection events by facility during the control or intervention periods

eFigure 4. Incidence of respiratory infections in PETRA in response to changes in facility infection control practices

eTable 3. Cumulative incidence of respiratory infections in control versus intervention conditions

eTable 4. Number of infections and incidence rate ratio (IRR) for acute respiratory infections after sensitivity adjustments

eTable 5. Number of infections and incidence rate ratio (IRR) for acute respiratory infections by zone after sensitivity adjustments

eFigure 5. Cumulative incidence of respiratory infections in control versus intervention after sensitivity adjustments and exclusions

eTable 6. Estimated increase in infections per week for residents in intervention versus control groups after sensitivity adjustments and exclusions

eTable 7. Incidence of public hospitalisation for complications associated with respiratory infection in intervention versus control groups

This supplemental material has been provided by the authors to give readers additional information about their work.

© 2025 Shoubridge AP et al. *JAMA Internal Medicine*.

eMethods

Trial design

Events external to the study necessitated modifications to the original trial design [1]. These included the Suez Canal obstruction in March 2021, which resulted in significant delays in the importation and subsequent installation of the GUV light infrastructure. In addition, South Australia (SA) state borders closed on 24 March, 2020, and reopened on 23 November, 2021 (**eFigure 2A**) [2,3]. The border closure allowed SA to pursue a COVID-19 eradication strategy, in contrast to the containment strategies employed elsewhere in Australia. As a consequence, SA was effectively free of SARS-CoV-2 virus circulation, apart from sporadic outbreaks, for a considerable portion of the study (**eFigure 2)**. In addition, border closures and infection control measures contributed to a substantially reduced burden of seasonal respiratory infections in the community, including those that commonly cause influenzae-like illness in LTCFs, such as RSV, rhinovirus, and influenza (**eFigure 2B**). In response, the study design was increased to seven cycles (continuous for the study duration), encompassing two full winter respiratory virus seasons.

Respiratory virus particle load within the air column and on LTCF fomites were secondary outcomes in the original trial protocol. However, COVID-19-related public health restrictions greatly reduced access to facilities and consequently, assessment of airborne respiratory virus carriage was, pragmatically, not undertaken.

Participants

LTCFs within metropolitan and regional South Australia were recruited pragmatically if they were able to sub-divide communal living areas into discrete areas (zones) that enabled a concurrent comparison of interventions in cohorts that were otherwise subject to the same facility infection control practices (e.g. environmental cleaning, staffing, and social distancing). LTCFs were also assessed for suitability and layout to enable the installation of GUV devices. Assessments were based on total resident populations, with no exclusion for age, sex, or other characteristics. Four LTCFs in South Australia participated in the cluster randomised trial (**eFigure 1**), each providing two discrete matched zones (n=8).

Interventions

UV-FLOW-C units (a high-output UV-C germicidal device) were bracketed to walls or ceilings, as appropriate. These units emit UV ray parallel beams (253.7nm wavelength), generated through reflection from a mirror-polished aluminium surface, which pass through a black honeycomb laminated grid to generate a unidirectional flow across the upper airspace. The germicidal zone, defined as areas with UV $\geq 10\mu W/cm^2$, extends approximately 5m from the unit. UV-FAN M2/95HP units are fan-driven wall-mounted or portable GUV devices that operate by drawing air from the room into an internal mirror-polished aluminium box containing two 95W, ozone-free, 253.7nm UV-C lamps, with sterilised air recirculated into the room. Two nanostructured titanium dioxide filters within the box act as a photo-catalyst by degrading organic and inorganic material. The UV-FAN M2/95HP operates with an air flow of $150m^3$/hour, effectively irradiating an area of $40m^2$. UV-FAN-XS devices operate in a similar manner to the UV-FAN M2/95HP devices, with an air flow of $70m^3$/hour. Reduced operational noise made these units more appropriate when located close to resident rooms.

Outcomes

ARI case definition reflected both national (Communicable Disease Network Australia) and local (Communicable Diseases Control Branch of South Australia) guidelines [4,5], which themselves were based on definitions established within the European Centre for Disease Prevention and Control guidelines [6]. The definition reflects the principal triggers for clinical referral and infection control, which were often initiated in the absence of pathology results, and the fact that collection of clinical samples for viral detection was not performed in all instances, particularly where individuals had significant cognitive impairment.

Identification of ARIs was performed by the same health professionals in intervention and control cohorts within each facility, and these health professionals were blinded to the study schedule. Atypical symptoms common in aged care populations (recent onset or increases in confusion, changes in baseline behaviour, falling, exacerbation of underlying chronic illness, such as increased shortness of breath in someone with congestive heart failure) were also taken into consideration.

Randomisation: Sequence generation

Zones were paired within facilities and randomised to intervention or control condition respectively for the first cycle. Concealed random allocation from a computer-generated random numbers table was used to determine the initial control/intervention order within the crossover design. Each zone represented the unit of randomisation and the level of intervention delivery.

Statistical methods
Descriptive analysis was performed using means and standard deviations for normally distributed variables, medians and interquartile ranges (IQR) for non-normally distributed variables, and frequencies (percentage) for categorical variables.
Due to the extended nature of the study that lasted a total of 110 weeks between the beginning of the first cycle and the end of the last cycle, we also assessed the rate of increase of the cumulative number of infections using time-series regression, and the difference in the increase by experimental condition. Autoregressive (AR) modelling was used to remove the autocorrelation in residuals from an ordinary least-squares (OLS) regression model by adding appropriate autocorrelation parameters. Stationarity of the time-series was then assessed for OLS regression and AR models using the Durbin-Watson (DW) test for autocorrelation and generation of autocorrelation function (ACF) plots, partial autocorrelation (PACF) plots, white noise series plots, standardised residual plots and residual normality plots. For the DW test, the null hypothesis of a unit root and stationarity of the time series (no autocorrelation) was rejected if $p<0.05$. The autoregressive model was formulated as:

$$y_t = x_t'\beta + v_t$$
$$v_t = -\varphi_1 v_{t-1} - \varphi_2 v_{t-2} - \cdots - \varphi_m v_{t-m} + \varepsilon_t$$

$\varepsilon_t \sim \text{IN}(0, \sigma^2)$, which indicates that each ε_t is independently and normally distributed with mean 0 and variance σ^2. The presented difference in infections is the direct comparison at the final timepoint.

Statistical software
Mixed effects Poisson regression was performed in Stata (Release 17. College Station, TX: StataCorp LLC) using the 'mepossion' command. Time-series analysis was performed in SAS (Release 3.81, SAS Institute, Cary NC) using the 'PROC TIMESERIES' and 'PROC AUTOREG' procedures. Statistical significance for all hypothesis testing was set using a 2-sided type 1 error rate of $\alpha=0.05$.

eReferences

1. Orkin AM, Gill PJ, Ghersi D, et al. Guidelines for Reporting Trial Protocols and Completed Trials Modified Due to the COVID-19 Pandemic and Other Extenuating Circumstances: The CONSERVE 2021 Statement. *JAMA*. Jul 20 2021;326(3):257-265. doi:10.1001/jama.2021.9941
2. Promote, Protect, Prevent, Progress – The Chief Public Health Officer's Report 2020-2022. South Australian Government. 13/12/2024, 2024. https://www.sahealth.sa.gov.au/wps/wcm/connect/public+content/sa+health+internet/about+us/publications+and+resources/reports/the+chief+public+health+officers+report/the+chief+public+health+officers+report
3. Commonwealth COVID-19 Response Inquiry Report. Department of the Prime Minister and Cabinet. 13/12/2024, 2024. https://www.pmc.gov.au/sites/default/files/resource/download/covid-19-response-inquiry-report.pdf
4. Guidelines for the Prevention, Control and Public Health Management of Influenza Outbreaks in Residential Care Facilities in Australia. Australian Federal Department of Health. 2017. https://www1.health.gov.au/inter net/main/publishing.nsf/Content/cdna-flu-guidelines.htm.
5. Infectious disease control. Government of South Australia; SA Health. 2017. https://www.sahealth.sa.gov.au/wps/wcm/connect/public+content/sa+health+internet/clinical+resources/clinical+programs+and+practice+guidelines/infectious+disease+control
6. Prevention and control of outbreaks of seasonal influenza in long-term facilities: a review of the evidence and best-practice guidance. World Health Organization; Regional Office for Europe. https://www.euro.who.int/__data/assets/pdf_file/0015/330225/LTCF-best-practice-guidance.pdf

© 2025 Shoubridge AP et al. *JAMA Internal Medicine*.

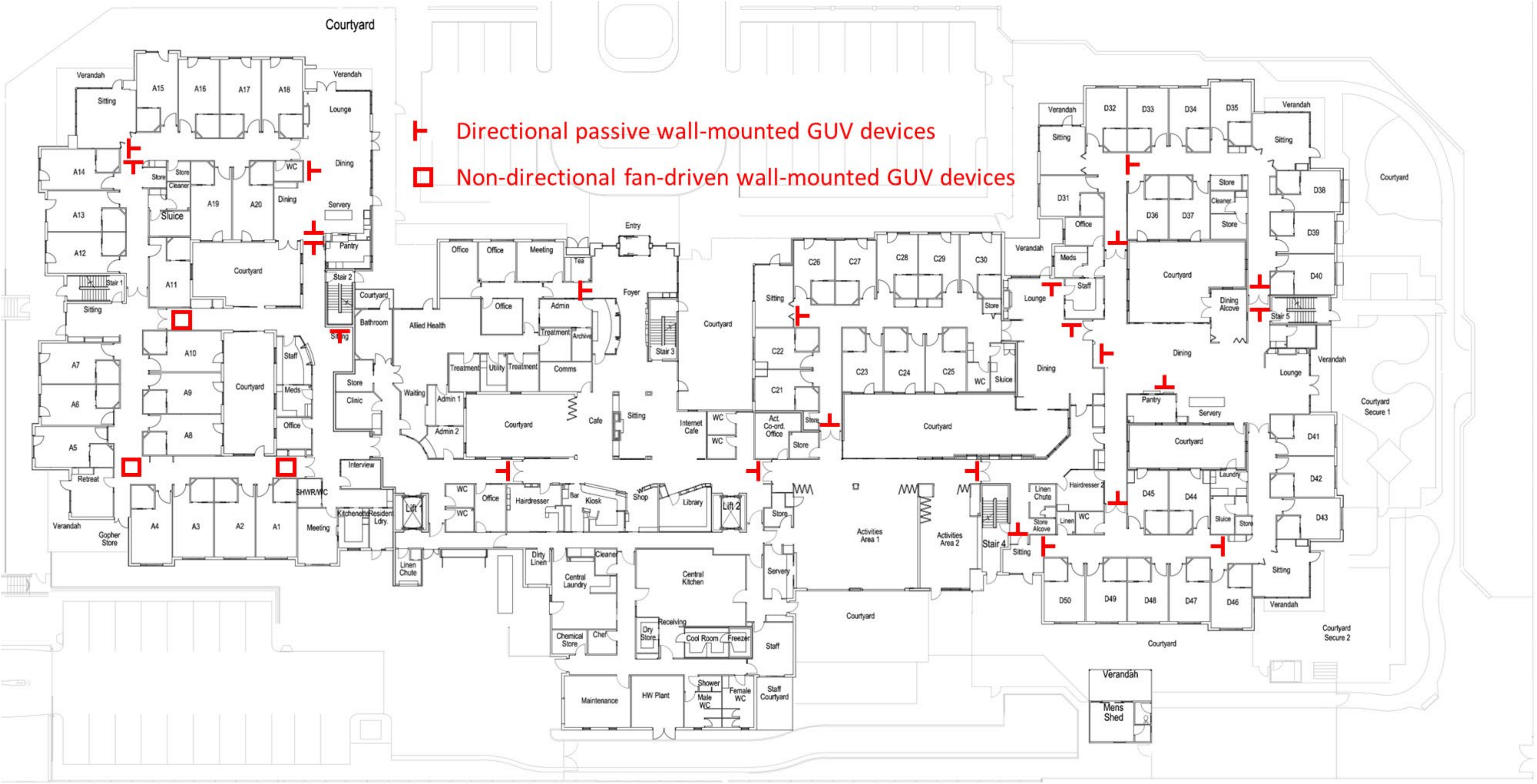

eFigure 1. Example deployment of GUV appliances across a LTCF zone.

This figure represents the layout of GUV appliances within a zone in a participating LTCF. An identical deployment pattern was used in the paired zone within the same facility. Residents commonly dine together in shared areas within their zone, and also congregate for group recreational activities. When not occupying these spaces, residents spend a substantial portion of their time residing within their private room. Opportunities for airborne pathogen transmission are therefore particularly great during mealtimes, and when bioaerosols generated within resident rooms migrate into shared corridor and connective spaces. The layout differed for each facility due to differences in building characteristics, such as layout and occupancy. Facility staff and technological engineers advised in the deployment of GUV appliances in a pattern for each individual facility that would optimally decontaminate the air of connecting corridors and spaces between resident rooms, while also providing coverage in high-traffic spaces (such as adjacency to lifts).

© 2025 Shoubridge AP et al. *JAMA Internal Medicine*.

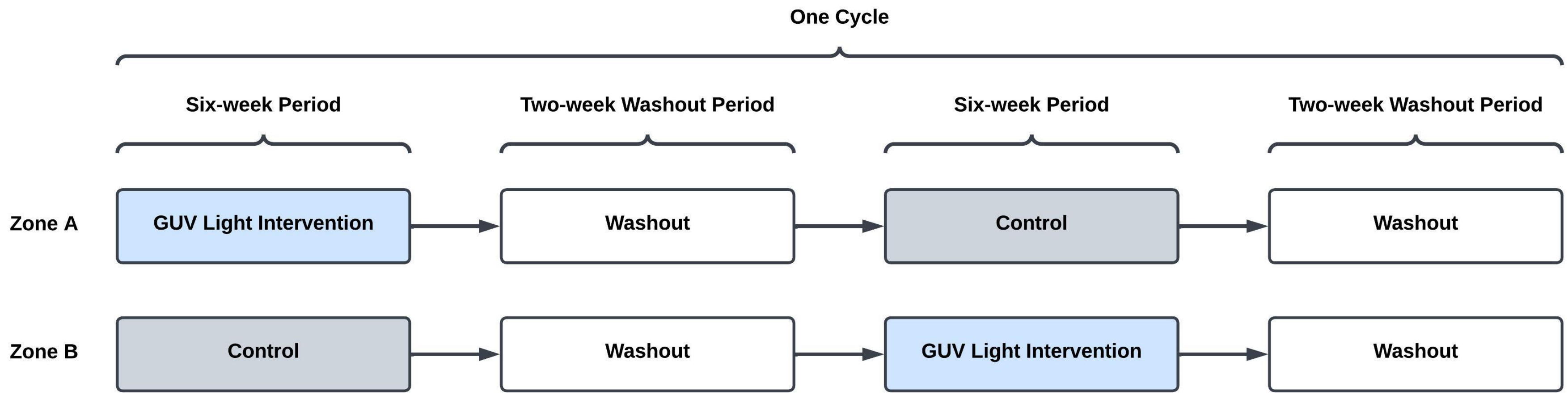

eFigure 2. PETRA trial stages.
Designated zones within LTCFs were paired, with one zone in each pair randomised to the intervention or control condition for the first six-week period. GUV appliances were switched off during control periods and run continuously during intervention periods. Six-week periods were divided by 2-week washout periods to account for viral incubation periods, before crossover to the reciprocal condition.

© 2025 Shoubridge AP et al. *JAMA Internal Medicine*.

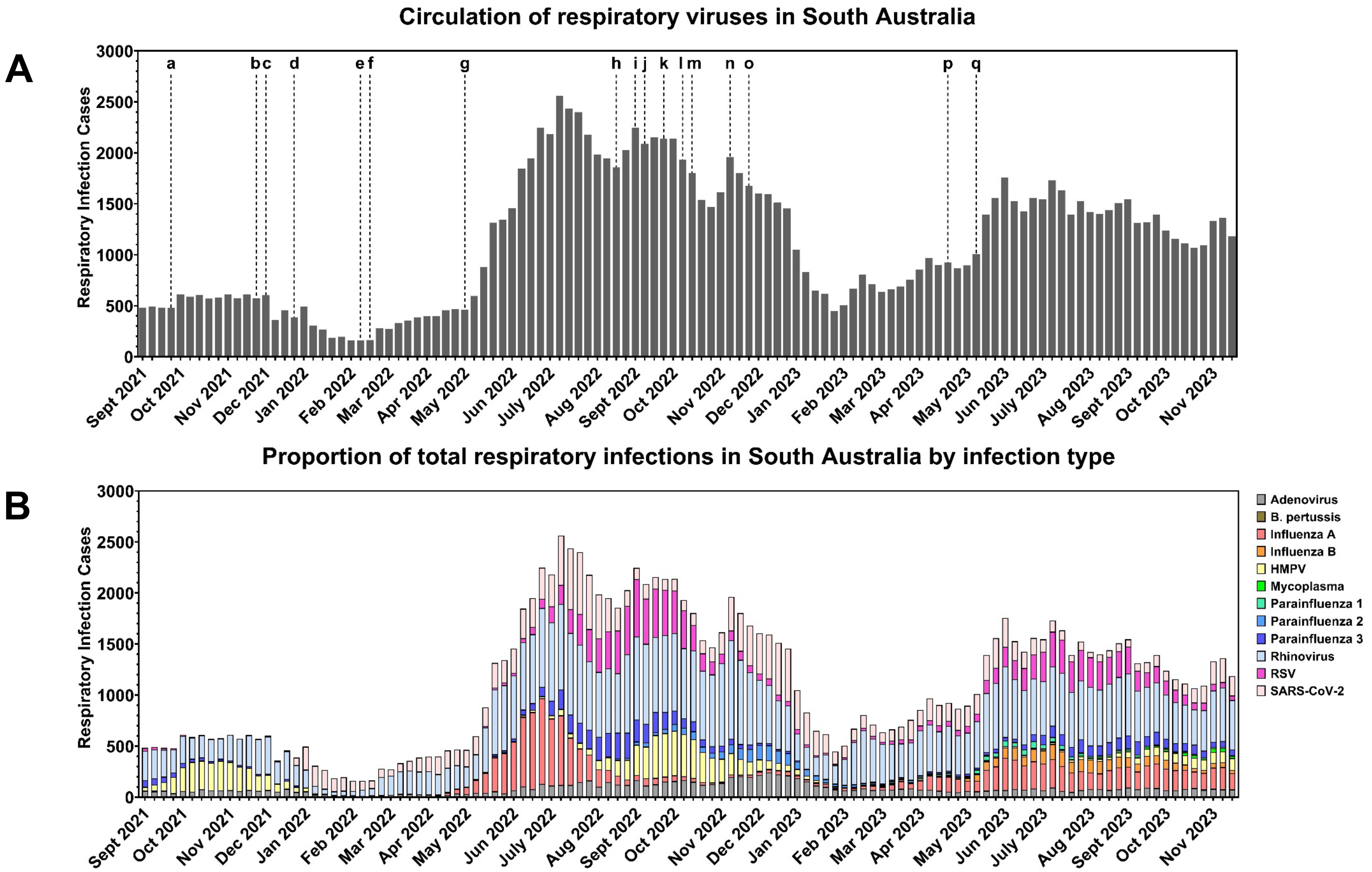

© 2025 Shoubridge AP et al. *JAMA Internal Medicine*.

eFigure 3. Respiratory virus circulation in South Australia (SA) corresponding with changes to national and state-wide changes in infection control practices.

A) Annotations correspond with the following nation-wide and state-wide changes to infection control practices during the PETRA timeline: **a)** National COVID-19 vaccinations reach 80%, **b)** State borders open within Australia, **c)** SA LTCF visitors required to be COVID-19 double-vaccinated, **d)** RATs approved in SA, **e)** Schools completely open in SA, **f)** National borders open for international travel, **g)** Home isolation lifted nationwide, **h)** LTCF resident isolation measures eased nationwide, **i)** LTCF screening eased in SA, **j)** Face mask mandate removed on domestic flights nationwide, **k)** LTCF visitor vaccinations lifted in SA, **l)** Home isolation lifted nationwide, **m)** Face mask use lifted in SA, **n)** Face mask use re-introduced (4-weeks) in SA, **o)** COVID-19 emergency declaration lifted in SA, **p)** LTCF staff influenza vaccination mandate lifted in SA, **q)** LTCF staff all vaccination mandates lifted nationwide. **B)** Respiratory infection proportion is estimated from results of South Australian samples tested by the SA Pathology respiratory viral multiplex PCR panel including the following pathogens: adenovirus, *B. pertussis*, influenza A, influenza B, HMPV, mycoplasma, parainfluenza 1, parainfluenza 2, parainfluenza 3, RSV, or SARS-CoV-2.

eTable 1. Baseline infection control practices of enrolled long-term aged care facilities at commencement of PETRA.

Baseline infection control practice	Facility 1	Facility 2	Facility 3	Facility 4
Staff with season's influenza vaccination, %	98	100	100	100
Staff with one dose COVID-19 vaccination, %	15	27	7	1
Staff with two doses COVID-19 vaccination, %	62	73	93	99
Surgical masks worn by staff	Yes	Yes	Yes	Yes
Face shields worn by staff	No	No	No	No
Restrictions on resident movements	No	Yes (density limitations of 1 person per 1.5m)	Yes (density limitations of 1 person per 1.5m)	Yes (density limitations of 1 person per 1.5m)
Visitation restrictions	No	No	No	Yes (visits between 1100AM-1500PM, maximum 4 visitors at 1 time)
Visitors screened (temperature and influenza vaccination)	Yes	Yes	Yes	Yes
Frequency of touch-points disinfected	Daily	2-3 times per day	2-3 times per day	2-3 times per day
Residents with season's influenza vaccination, %	89	98	84	91
Residents with one dose COVID-19 vaccination, %	7	4	2	6
Residents with two doses COVID-19 vaccination, %	81	93	89	76

© 2025 Shoubridge AP et al. *JAMA Internal Medicine*.

eTable 2. Characteristics of residents with reported acute respiratory infection events by facility during the control or intervention periods.

Symptom onset, No. (%) (not mutually exclusive)	Facility 1	Facility 2	Facility 3	Facility 4	Total
Cough (new or worsening)	118 (45)	39 (51)	43 (47)	14 (30)	214 (45)
Sore throat	53 (20)	15 (20)	8 (9)	11 (24)	87 (18)
Shortness of breath	13 (5)	9 (12)	3 (3)	3 (7)	28 (6)
Fever or feverishness	26 (10)	2 (3)	6 (7)	3 (7)	37 (8)
Malaise	7 (3)	6 (8)	5 (5)	1 (2)	19 (4)
Headache	3 (1)	3 (4)	0 (0)	1 (2)	7 (1)
Myalgia	0 (0)	4 (5)	1 (1)	0 (0)	5 (1)
Runny or congested nose	64 (25)	16 (21)	29 (32)	9 (20)	118 (25)
Loss of taste or smell	0 (0)	1 (1)	0 (0)	0 (0)	1 (0)
Fatigue	12 (5)	3 (4)	9 (10)	2 (4)	26 (5)
Nausea, vomiting, or diarrhoea	5 (2)	1 (1)	0 (0)	2 (4)	8 (2)
Loss of appetite	0 (0)	1 (1)	0 (0)	0 (0)	1 (0)

© 2025 Shoubridge AP et al. *JAMA Internal Medicine*.

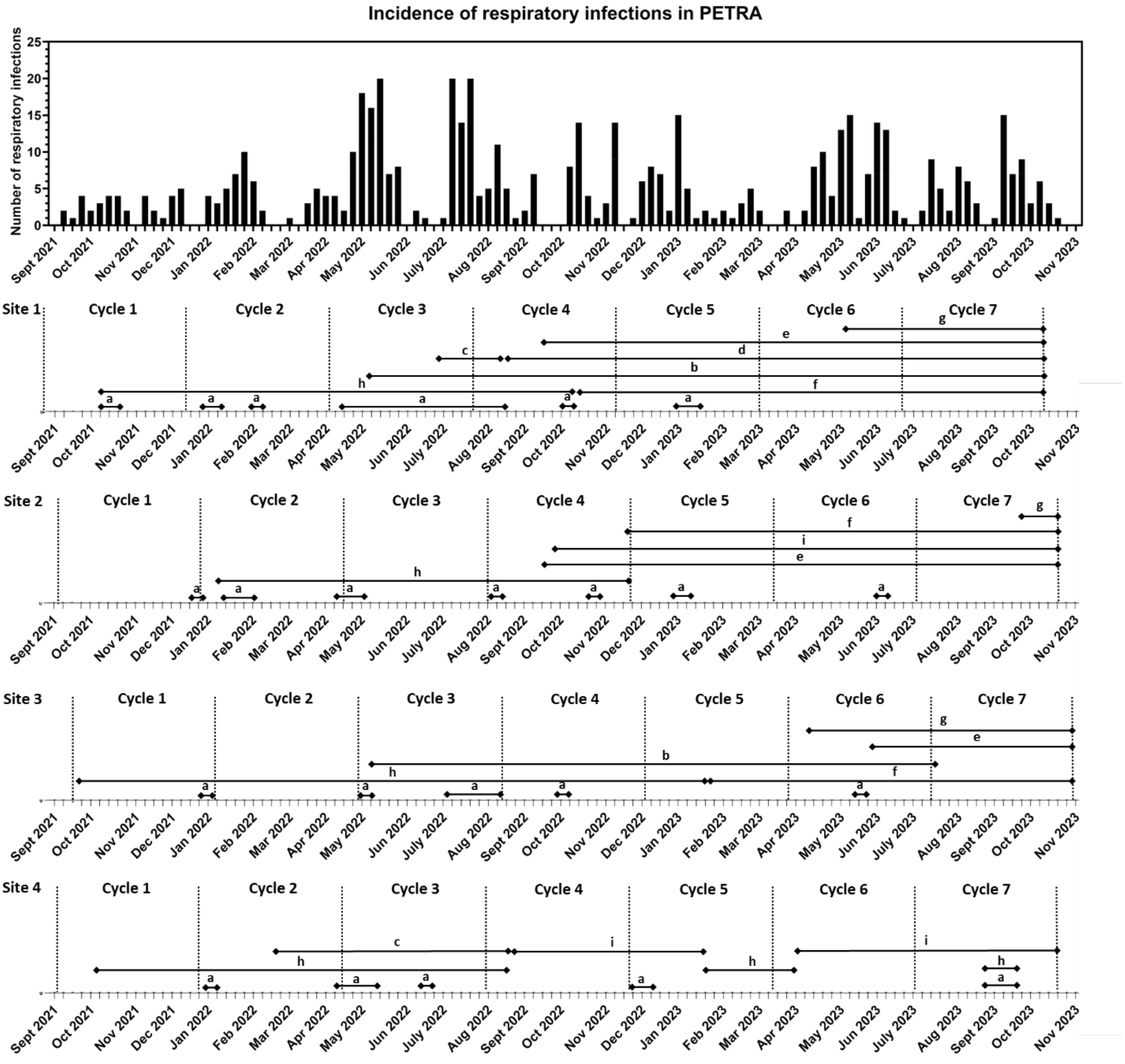

eFigure 4. Incidence of respiratory infections in PETRA in response to changes in facility infection control practices.

The number and distribution of ARI events across all LTCFs in PETRA from August 31, 2021, to November 13, 2023, with the corresponding timeline of facility-wide events for each individual LTCF. Annotations correspond with the following facility-level changes to infection control practices during the PETRA timeline: **a)** Lockdown of facility or zones within facilities that were involved in the study, **b)** All residents subjected to RAT testing for SARS-CoV-2 every 48 hours, **c)** All staff and visitors subjected to RAT testing for SARS-CoV-2 daily, **d)** All staff subjected to RAT testing for SARS-CoV-2 every 48 hours, **e)** No requirement for staff and visitors to be vaccinated against influenza and COVID-19, **f)** No requirement for staff and visitors to wear face masks, **g)** No requirement for isolation of symptomatic residents, **h)** Staff and visitors required to wear face masks, **i)** Visitors not required to undergo COVID-19 RAT testing and temperature screening.

© 2025 Shoubridge AP et al. *JAMA Internal Medicine*.

eTable 3. Cumulative incidence of respiratory infections in control versus intervention conditions. The cumulative incidence of respiratory infections of the control and intervention conditions over 110 consecutive weeks, including and excluding events in memory support units (MSU). Predicted series and predicted trends were calculated with autoregressive modelling after removing level-2 autocorrelation.

**Condition**	**Increase in infections per week β (95% CI)**	**Difference in infections (95% CI) per week (P-value: Intervention versus Control)**
**All beds**		
Control	2.61 (2.51 to 2.70)	-0.319 (-0.542 to -0.096) (p=0.004)
Intervention	2.29 (2.06 to 2.51)	
AR1[a]	-1.275 (-1.42 to -1.13)	p<0.001
AR2[b]	0.323 (0.18 to 0.47)	p<0.001
**Excluding MSU beds**		
Control	2.14 (2.04 to 2.24)	-0.406 (-0.603 to -0.209) (p<0.001)
Intervention	1.73 (1.54 to 1.92)	
AR1[a]	-1.19 (-1.34 to -1.04)	p<0.001
AR2[b]	0.24 (0.09 to 0.40)	p=0.001

[a] First order autoregressive parameters in autoregressive time-series analysis.
[b] Second order autoregressive parameters in autoregressive time-series analysis.

© 2025 Shoubridge AP et al. *JAMA Internal Medicine*.

eTable 4. Number of infections and incidence rate ratio (IRR) for acute respiratory infections after sensitivity adjustments.

Adjustment	Condition	Recorded events (n)	Infections per zone per cycle Mean (95% CI)	Infections per 1000 bed-days Mean (95% CI)	Incidence rate ratio[1] (95% CI)	Estimated mean difference in infections per zone per cycle (95% CI) (Intervention vs Control)	P-value[1]
Three-day incubation window	Control	249	4.14 (2.40, 5.89)	2.38 (1.70, 3.06)	1.00 (Ref)	-0.29 (-1.01, 0.44)	0.433
	Intervention	232	3.86 (2.23, 5.49)	2.22 (0.51, 3.93)	0.93 (0.78, 1.11)		
MSU exclusion with three-day incubation window	Control	194	3.27 (1.88, 4.66)	1.85 (1.25, 2.45)	1.00 (Ref)	-0.18 (-0.82, 0.47)	0.584
	Intervention	184	3.09 (1.77, 4.41)	1.76 (0.83, 2.69)	0.95 (0.77, 1.16)		

[1]From mixed effects Poisson regression model with zone as a random effect.

© 2025 Shoubridge AP et al. *JAMA Internal Medicine*.

eTable 5. Number of infections and incidence rate ratio (IRR) for acute respiratory infections by zone after sensitivity adjustments.

Adjustment	Condition	Group	Recorded events (n)	Observed Infections per zone per cycle Mean (95% CI)	Estimated mean difference in infections per zone per cycle (95% CI)[2] (Intervention vs Control)	Infections per 1000 bed-days Mean (95% CI)	Incidence rate ratio[1] (95% CI)	P-value[3]
None	Facility 1 Zone A	Control	65	9.3 (2.2, 16.3)	-0.98 (-3.53, 1.58)	4.0 (0.9, 7.1)	0.87 (0.61, 1.25)	0.454
		Intervention	56	8.0 (-2.2, 18.2)		3.5 (-1.0, 8.0)		
	Facility 1 Zone B	Control	86	12.3 (8.0, 16.6)	-3.86 (-6.86, -0.86)	5.4 (3.5, 7.3)	0.62 (0.44, 0.87)	0.011
		Intervention	54	7.7 (3.7, 11.7)		3.4 (1.6, 5.1)		
	Facility 2 Zone A	Control	14	2.0 (0.5, 3.5)	2.29 (-0.04, 4.62)	1.3 (0.4, 2.2)	1.93 (1.01, 3.68)	0.054
		Intervention	27	3.9 (1.0, 6.7)		2.5 (0.6, 4.4)		
	Facility 2 Zone B	Control	24	3.4 (1.2, 5.6)	-2.29 (-4.46, -0.12)	2.2 (0.8, 3.6)	0.46 (0.22, 0.94)	0.039
		Intervention	11	1.6 (0.5, 2.7)		1.0 (0.3, 1.8)		
	Facility 3 Zone A	Control	15	2.1 (-1.0, 5.2)	2.32 (0.52, 4.12)	1.0 (-0.4, 2.4)	2.29 (1.25, 4.19)	0.010
		Intervention	35	5.0 (-1.9, 11.9)		2.2 (-0.8, 5.2)		
	Facility 3 Zone B	Control	12	1.7 (0.2, 3.2)	2.19 (0.52, 3.86)	0.7 (0.1, 1.4)	2.54 (1.31, 4.98)	0.011
		Intervention	30	4.3 (-1.6, 10.2)		1.9 (-0.8, 4.7)		
	Facility 4 Zone A	Control	25	3.6 (0.3, 6.8)	-3.13 (-5.43, -0.84)	2.5 (0.2, 4.7)	0.32 (0.14, 0.71)	0.008
		Intervention	8	1.1 (0.2, 2.0)		0.8 (0.2, 1.4)		
	Facility 4 Zone B	Control	7	1.0 (-0.1, 2.1)	-0.18 (-1.47, 1.12)	0.7 (-0.1, 1.5)	0.86 (0.29, 2.57)	0.079
		Intervention	6	0.9 (0.0, 1.8)		0.6 (0.0, 1.2)		
Three-day incubation window	Facility 1 Zone A	Control	68	9.7 (2.5, 16.9)	-1.32 (-3.92, 1.28)	4.2 (1.1, 7.4)	0.83 (0.59, 1.19)	0.318
		Intervention	56	8.0 (-2.2, 18.2)		3.5 (-1.0, 8.0)		
	Facility 1 Zone B	Control	88	12.6 (8.2, 16.9)	-3.75 (-6.77, -0.72)	5.5 (3.6, 7.4)	0.64 (0.46, 0.89)	0.015
		Intervention	57	8.1 (3.8, 12.5)		3.5 (1.7, 5.4)		
	Facility 2 Zone A	Control	13	1.9 (0.6. 3.1)	2.46 (0.15, 4.78)	1.2 (0.4, 2.0)	2.07 (1.07, 4.02)	0.037
		Intervention	27	3.9 (1.3, 6.4)		2.5 (0.9, 4.2)		
	Facility 2 Zone B	Control	24	3.4 (1.1, 5.6)	-2.29 (-4.46, -0.12)	2.2 (0.8, 3.6)	0.46 (0.22, 0.94)	0.039
		Intervention	11	1.6 (0.4, 2.7)		1.0 (0.3, 1.8)		
	Facility 3 Zone A	Control	16	2.3 (-1.1, 5.6)	2.20 (0.40, 4.00)	1.0 (-0.5, 2.6)	2.14 (1.19, 3.88)	0.017
		Intervention	35	5.0 (-1.9, 11.9)		2.2 (-0.8, 5.2)		

© 2025 Shoubridge AP et al. *JAMA Internal Medicine*.

	Facility 3 Zone B	Control	12	1.7 (0.2, 3.2)	2.42 (0.69, 4.16)	0.7 (0.1, 1.4)	2.72 (1.40, 5.28)	0.006
		Intervention	32	4.6 (-1.2, 10.4)		2.1 (-0.6, 4.7)		
	Facility 4 Zone A	Control	20	2.9 (0.3, 5.4)	-2.21 (-4.24, -0.18)	2.0 (0.2, 3.7)	0.40 (0.18, 0.90)	0.032
		Intervention	8	1.1 (0.2, 2.0)		0.8 (0.2, 1.4)		
	Facility 4 Zone B	Control	8	1.1 (0.1, 2.2)	-0.36 (-1.71, 0.99)	0.8 (0.0, 1.2)	0.75 (0.26, 2.17)	0.602
		Intervention	6	0.9 (-0.0, 1.8)		0.6 (0.0, 1.2)		

[1]Ratio of infections per 1000 bed days for Group 2 versus Group 1 from mixed effects Poisson regression model with cycle as a random effect.
[2]Estimated difference in mean infections for Group 2 versus Group 1 from mixed effects Poisson regression model with cycle as a random effect.
[3]P-value for estimated difference in number of infections per zone per cycle.

© 2025 Shoubridge AP et al. *JAMA Internal Medicine*.

A

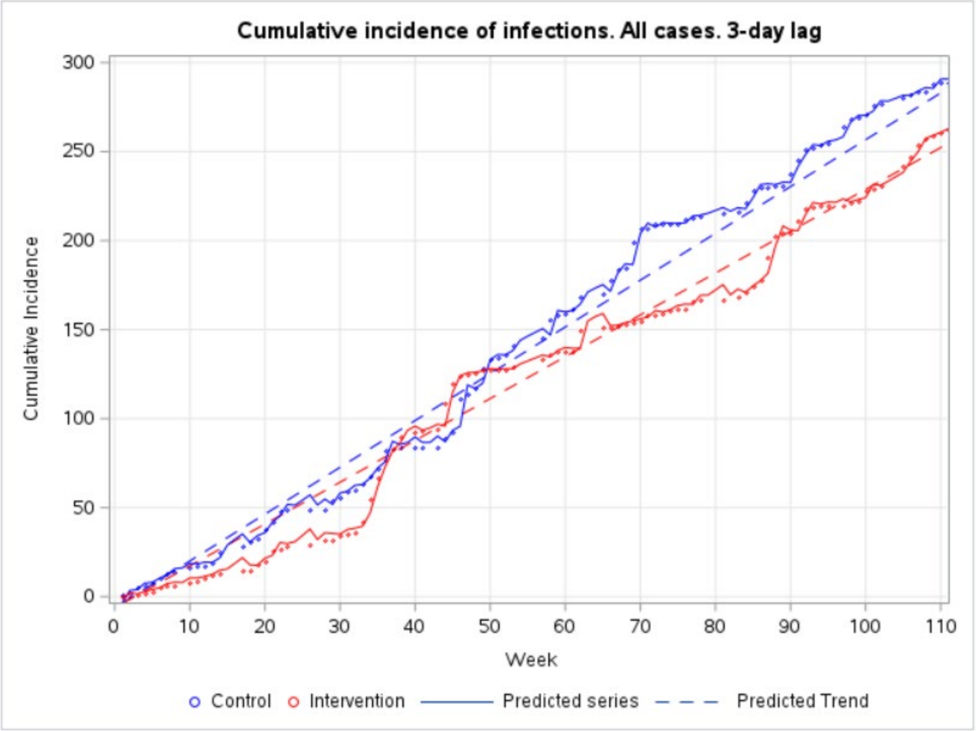

B

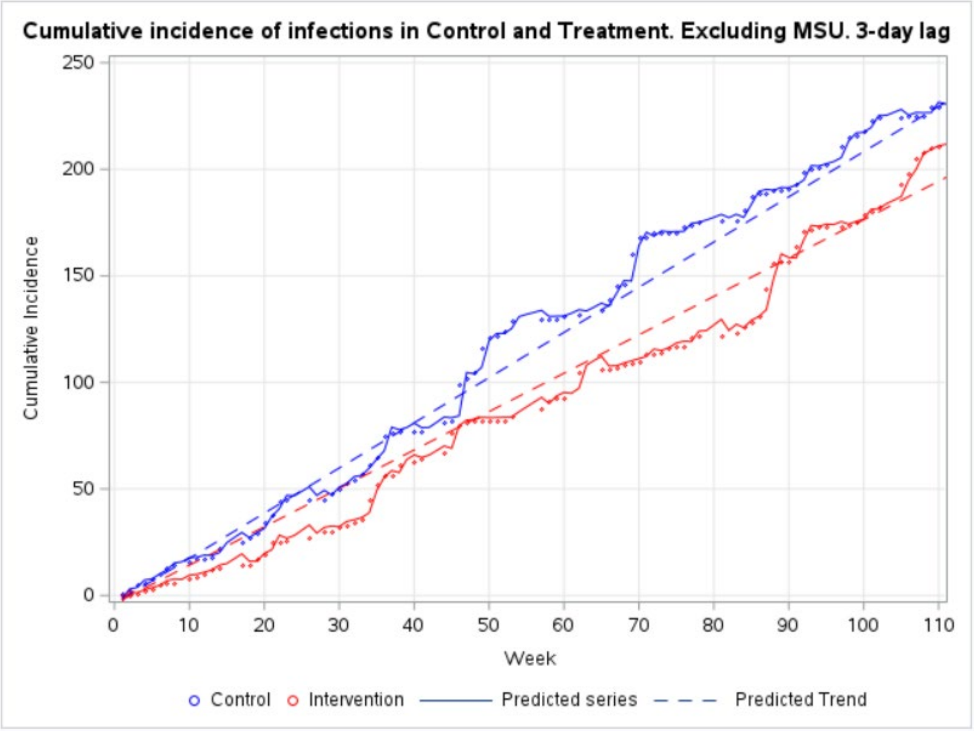

eFigure 5. Cumulative incidence of respiratory infections in control versus intervention after sensitivity adjustments and exclusions.

The observed and predicted cumulative incidence of respiratory infections of the control and intervention conditions over 110 consecutive weeks. Predicted series and predicted trends were calculated with autoregressive modelling after removing level-2 autocorrelation. **A)** Adjusting for a three-day incubation window. **B)** Adjusting for a three-day lag window and excluding events that occurred in MSUs.

© 2025 Shoubridge AP et al. *JAMA Internal Medicine*.

eTable 6. Estimated increase in infections per week for residents in intervention versus control groups after sensitivity adjustments and exclusions.

Adjustment	Condition	Increase in infections per week β (SE)	Difference in slopes (SE) (Δ cases per week) (P-value: Intervention versus Control)
Three-day incubation window	Control	2.63 (0.046)	-0.279 (0.107) (p=0.010)
	Intervention	2.35 (0.107)	
	AR1	-1.263	-
	AR2	0.311	-
MSU exclusion with three-day incubation window	Control	2.12 (0.049)	-0.319 (0.104) (p=0.002)
	Intervention	1.80 (0.093)	
	AR1	-1.204 (0.075)	-
	AR2	0.253 (0.076)	-

AR1, AR2=Autoregressive parameters.

© 2025 Shoubridge AP et al. *JAMA Internal Medicine*.

eTable 7. Incidence of public hospitalisation for complications associated with respiratory infection in intervention versus control groups.

Condition	Recorded events (n)
Control	13
Intervention	9

Note: Data relate to hospitalisations only accessible for those treated within the public hospital system. Hospitalisation data for residents admitted via the private sector were inaccessible.

© 2025 Shoubridge AP et al. *JAMA Internal Medicine*.
