# Supplementary material for: Germicidal UV Light and Incidence of Acute Respiratory Infection in Long-Term Care for Older Adults: A Randomized Clinical Trial
Source: JAMA Intern Med. 2025 Jul 28;185(9):1128–35. doi: 10.1001/jamainternmed.2025.3388 (PMC12305439; doi:10.1001/jamainternmed.2025.3388)
Supplement: Supplement 4. — Data sharing statement [file jamainternmed-e253388-s004.pdf]

Data Sharing Statement

Shoubridge. Germicidal UV Light and Incidence of Acute Respiratory Infection in Long-Term Care for Older Adults. *JAMA Intern Med*. Published July 28, 2025.
doi:10.1001/jamainternmed.2025.3388

Data
Additional Information: Australian and New Zealand Clinical Trial Registration number ACTRN12621000567820 (https://anzctr.org.au/).
Data available: Yes
Data types: Deidentified participant data, Data dictionary
How to access data: Data and statistical code will be made available upon request to the corresponding author, Prof Geraint Rogers (geraint.rogers@sahmri.com).
When available: With publication

Supporting Documents
Document types: Statistical/analytic code
How to access documents: Data and statistical code will be made available upon request to the corresponding author, Prof Geraint Rogers (geraint.rogers@sahmri.com).
When available: With publication

Additional Information
Who can access the data: Researchers whose proposed use of the data has been approved.
Types of analyses: For any purpose.
Mechanisms of data availability: After approval of a proposal.
